# Lysosomal dysfunction and overload of nucleosides in thymidine phosphorylase deficiency of MNGIE

**DOI:** 10.1186/s12967-024-05275-8

**Published:** 2024-05-13

**Authors:** Jixiang Du, Fuchen Liu, Xihan Liu, Dandan Zhao, Dongdong Wang, Hongsheng Sun, Chuanzhu Yan, Yuying Zhao

**Affiliations:** 1 Department of Rheumatology and Immunology, Shandong Provincial Hospital Affiliated to Shandong First Medical University, Jinan, 250021 Shandong China; 2 Research Institute of Neuromuscular and Neurodegenerative Disease, Department of Neurology, Cheeloo College of Medicine, Qilu Hospital, Shandong University, West Wenhua Street No.107, Jinan, 250012 Shandong China; 3 Department of Rheumatology and Immunology, Cheeloo College of Medicine, Shandong Provincial Hospital, Shandong University, Jinan, 250021 Shandong China; 4 Key Laboratory of Experimental Teratology, Ministry of Education, School of Basic Medical Science, Department of Obstetrics and Gynecology, Qilu Hospital, Shandong University, Jinan, 250012 Shandong China; 5 Mitochondrial Medicine Laboratory, Qilu Hospital (Qingdao), Cheeloo College of Medicine, Shandong University, Qingdao, 266000 Shandong China; 6 Brain Science Research Institute, Shandong University, Jinan, 250012 Shandong China

**Keywords:** TYMP, Thymidine phosphorylase, MNGIE, Lysosomal dysfunction, Nucleotide metabolism

## Abstract

Inherited deficiency of thymidine phosphorylase (TP), encoded by *TYMP*, leads to a rare disease with multiple mitochondrial DNA (mtDNA) abnormalities, mitochondrial neurogastrointestinal encephalomyopathy (MNGIE). However, the impact of TP deficiency on lysosomes remains unclear, which are important for mitochondrial quality control and nucleic acid metabolism. Muscle biopsy tissue and skin fibroblasts from MNGIE patients, patients with m.3243 A > G mitochondrial encephalopathy, lactic acidosis and stroke-like episodes (MELAS) and healthy controls (HC) were collected to perform mitochondrial and lysosomal functional analyses. In addition to mtDNA abnormalities, compared to controls distinctively reduced expression of LAMP1 and increased mitochondrial content were detected in the muscle tissue of MNGIE patients. Skin fibroblasts from MNGIE patients showed decreased expression of LAMP2, lowered lysosomal acidity, reduced enzyme activity and impaired protein degradation ability. *TYMP* knockout or TP inhibition in cells can also induce the similar lysosomal dysfunction. Using lysosome immunoprecipitation (Lyso- IP), increased mitochondrial proteins, decreased vesicular proteins and V-ATPase enzymes, and accumulation of various nucleosides were detected in lysosomes with TP deficiency. Treatment of cells with high concentrations of dThd and dUrd also triggers lysosomal dysfunction and disruption of mitochondrial homeostasis. Therefore, the results provided evidence that TP deficiency leads to nucleoside accumulation in lysosomes and lysosomal dysfunction, revealing the widespread disruption of organelles underlying MNGIE.

**Graphical Abstract:**

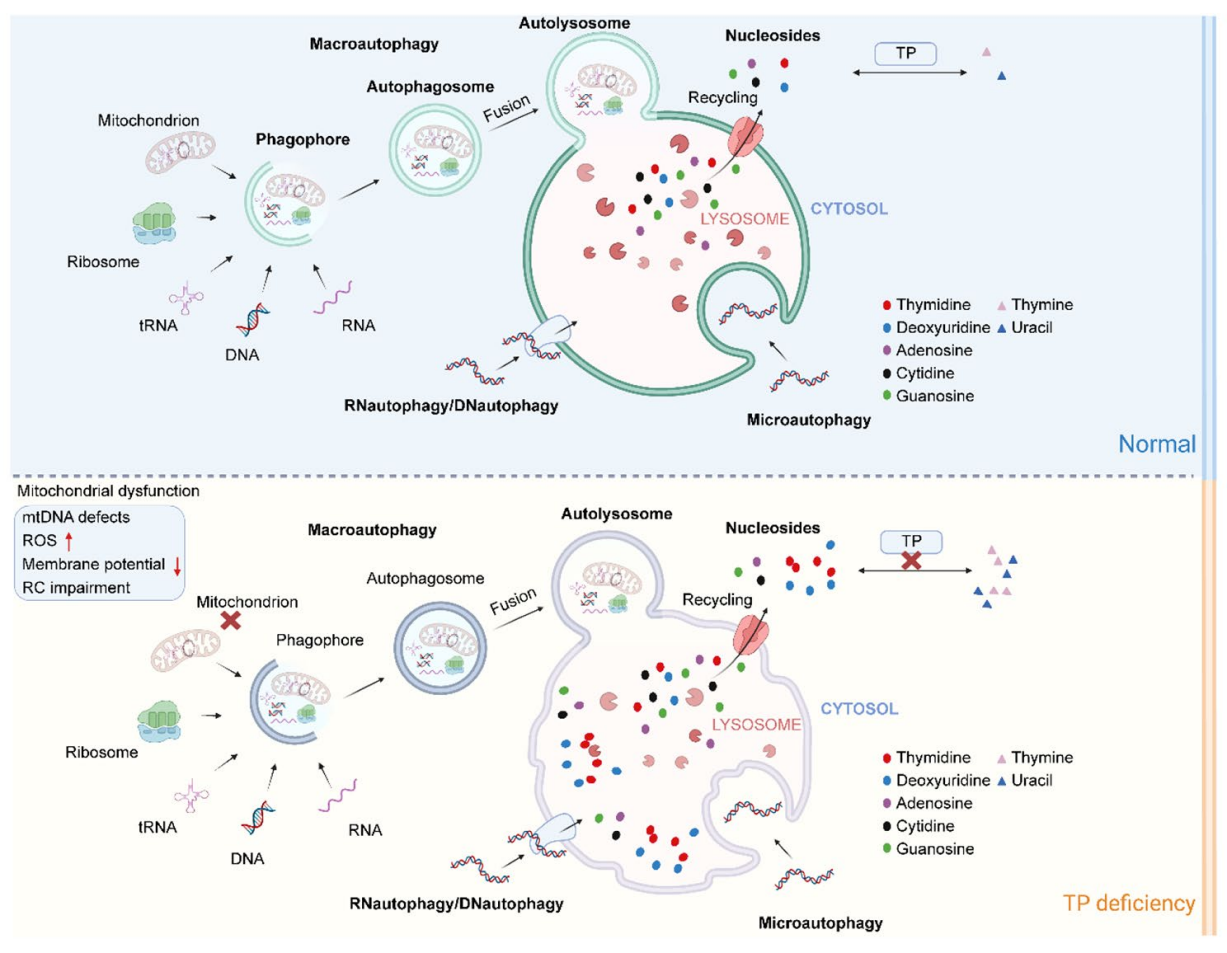

**Supplementary Information:**

The online version contains supplementary material available at 10.1186/s12967-024-05275-8.

## Introduction

TP, encoded by *TYMP*, is a cytosolic metabolic enzyme that degrades thymidine (dThd) or deoxyuridine (dUrd) to thymine or uracil. Inherited deficiency of *TYMP* causes an autosomal recessive disease with mtDNA abnormalities called MNGIE, which is a rare, progressive and fatal disease that mainly affects the gastrointestinal and neurological systems [1]. Previous research has suggested that the mtDNA defects in MNGIE are due to an imbalance of the mitochondrial nucleotide pool caused by mitochondrial accumulation of dThd/dUrd and corresponding nucleotides [2]. Unlike primary mitochondrial diseases, which are caused by defects in mitochondrial proteins encoded by nuclear or mitochondrial genes, the nature of MNGIE is an inherited metabolic disorder caused by a deficiency of the cytoplasmic enzyme TP. Previous research on TP suggests that TP has several functions, including promoting angiogenesis, regulating cell growth and proliferation, inhibiting apoptosis, regulating platelet activation and controlling adipocyte differentiation. However, the mechanisms of action of TP are not yet fully understood [3–11]. Therefore, in addition to influencing mtDNA replication, TP may also be involved in other cellular activities to exert its effect. Therefore, the pathological mechanisms underlying the clinical phenotype of MNGIE due to TP deficiency are incompletely understood. In particular, the effects of intracellular nucleoside accumulation and TP protein deficiency on other organelles such as the endoplasmic reticulum, lysosomes and cytoskeleton are still unclear.

As a cellular “recycling center” and hub for metabolic signals, lysosomes are closely involved in mitochondrial quality control. On the one hand, lysosomes play a crucial role in mitochondrial clearance, including various pathways such as PINK1-parkin-, BNIP3- or FUNDC1-mediated mitophagy, mitochondria-derived vesicles, migrasome-mediated mitochondrial ejection and lysosome-associated exocytosis [12–20]. On the other hand, mitochondria and lysosomes can form dynamic membrane contact sites or connections for direct communication through proteins such as Rab7, TBC1D15, Fis1 and GDAP1 [21–24]. This interaction between mitochondria and lysosomes plays a crucial role in regulating the dynamics, function and metabolite transport of organelle networks [25–28]. Thus, there is a close structural and functional link between lysosomes and mitochondria, but the status of lysosomal function in MNGIE remains unclear.

TP is a key enzyme in nucleotide metabolism, while lysosomes play an important role in the degradation and recycling of nucleic acids. On the one hand, nucleic acids can be delivered to lysosomes as part of larger structures by mitochondrial autophagy, ribosomal autophagy, nuclear budding, piecemeal microautophagy of the nucleus, RNA granule autophagy, etc [29–34]. . On the other hand, RNA and DNA can be directly transported to lysosomes for degradation by receptors such as LAMP2C and SIDT2 in an ATP-dependent manner, a process known as RNA autophagy/DNA autophagy [35, 36]. Once various forms of nucleic acid molecules enter the lysosomal lumen as autophagic substrates, they are degraded into mononucleotides or oligonucleotides by acid hydrolases such as RNase T2 and DNase II [37]. The nucleotides are further hydrolyzed to nucleosides. Nucleosides, the end products of nucleic acid degradation in lysosomes, are transported back into the cytoplasm via lysosomal nucleoside transport proteins such as SLC29A3/ENT3. They are then degraded to the corresponding bases or enter the recycling pathways [38, 39].

Overall, lysosomes are important cellular organelles involved in the regulation of mitochondrial homeostasis and nucleotide metabolism. Elucidating the effects of TP defects on lysosomal function is crucial for further understanding the pathophysiological mechanisms of MNGIE. In this study, we identified a lysosomal dysfunction caused by *TYMP* deficiency that is associated with the accumulation of nucleosides in lysosomes.

## Materials and methods

### Participants and sample collection

The MNGIE and MELAS patients are the same patients reported in our previous study, with matching clinical information and sample names [40]. The muscle biopsies and punch biopsies of the skin were performed according to standard procedures. Patient MNGIE-2 declined the muscle biopsy, and the muscle biopsy of patient MNGIE-3 was completed by another hospital in his adolescence with a few remaining muscle samples.

For the skin biopsies in the HC group, the individuals and collection procedure were the same as in our previous study [40]. The muscle biopsies in the HC group were sex- and age-matched healthy control samples (no abnormalities were detected in the muscle biopsy).

### Cell culture, plasmids and cell line constructs

Fibroblasts were obtained from skin biopsies. 293T cells were purchased from the American Type Culture Collection (ATCC, CRL-3216). Cells were cultured in Dulbecco’s modified Eagle medium (DMEM; CM10013, MACGENE) supplemented with 10% fetal bovine serum (16,140,071, Gibco) and penicillin/streptomycin (P1400, Solarbio) at 37 ℃/5% CO2. Fibroblasts were grown to 80 to 90% confluence before harvesting and passaging, with no more than 20 passages. All cell lines tested negative for mycoplasma contamination.

For the knockout of *TYMP*, the pLentiCRISPR-sgTYMP plasmid was constructed using the vector pLentiCRISPRv2 and the sgRNA sequence published in a previous study (sense 5′ CAGAGATGTGACAGCCACCG 3′; antisense 5′ CGGTGGCTGTCACATCTCTG 3′). pLentiCRISPR-sgTYMP and the control plasmids were transfected onto 293T cells using Lipofectamine 2000 reagents (Invitrogen). After 24 h of transfection, cells were seeded at a density of 1 cell/well in 96-well plates and then single cell colonies were grown. The cell clones of *TYMP*-knocked out were validated by analyzing TP protein expression by Western blot.

For the overexpression of TMEM192-3×HA, the sequence of TMEM192-3×HA from pLJC5-TMEM192-3xHA (Addgene plasmid # 102,930) was incorporated into the pLVX-Puro vector. The pLVX-TMEM192-3xHA-Puro plasmid was cotransfected with psPAX2 and phCMV-VSV-G packaging vectors to obtain a lentivirus encoding TMEM192-3xHA. 293T cells were transduced with the TMEM192-3×HA lentivirus, selected with puromycin, and single cell clones were isolated to generate the stable TMEM192-3×HA expressing 293T cell line. Based on the stable TMEM192-3×HA expressing cells obtained, *TYMP* knockout was performed according to the procedure described above.

For the mCherry-EGFP-LC3 reporter, the sequence of mCherry-EGFP-LC3B was obtained from the pBabe-puro mCherry-EGFP-LC3B (Addgene plasmid #22,418) and cloned into the vector pLenti-CMV-IRES-Puro-WPRE. For lentiviral packaging, mCherry-EGFP-LC3B was co-transfected into 293T cells together with psPAX2 and phCMV-VSV-G packaging vectors using Lipofectamine 2000. Stable mCherry-EGFP-LC3 expressing 293T cells were generated by lentiviral transduction and selection in growth media containing 1 µg/mL puromycin.

### Western blot

The procedures for Western blotting have been described in our previous publications. In brief, the lysis buffer used for protein isolation of cells and tissues was radioimmunoprecipitation assay buffer (R0020, Solarbio) supplemented with protease inhibitors (HY-K0010, MCE) and phosphatase inhibitors (P1260, Solarbio). To increase the efficiency of lysis, the frozen muscle tissue were cut into 8 mm cross-sections using a cryostat, manually pulverized in liquid nitrogen and then mixed with the lysis buffer. A total of 10–20 µg of proteins were loaded onto 8–12% SDS-PAGE. After blocking with 5% milk at room temperature for 1 h, the membranes were incubated overnight with the primary antibody at 4 °C. The membranes were then washed with Tris-buffered saline with Tween 20 (TBST) buffer and incubated with horseradish peroxidase-linked antibody at room temperature for 1 h, followed by another round of TBST washes. Protein bands were detected using the Tanon 4600 SF chemiluminescence imaging system and grayscale values of the bands were calculated using ImageJ software. Supplementary Table 1 contains a list of the antibodies used. Uncropped original Western blots were uploaded in Supplementary file 9.

### Quantitative reverse transcription PCR and mtDNA copy number analysis

Total RNA was extracted from tissues and cells using TRIzol (15,596,026, Invitrogen). Prior to mixing with TRIzol, frozen muscle biopsy tissue were sectioned to 8 mm cross-section using a cryostat and manually pulverized in liquid nitrogen. Complementary DNA was synthesized from 1 µg of RNA with HiScript II Q RT SuperMix (R223-01, Vazyme). SYBR Green Master Mix (Q711-02, Vazyme) was used to perform quantitative PCR (qPCR) in the QuantStudio 3 Real-Time PCR System. The relative quantification of mRNA expression was normalized to the reference gene ACTB.

Genomic DNA from muscle tissues and cells were isolated with the TIANamp Genomic DNA Kit (TIANGEN, DP304). Relative mtDNA copy numbers were analyzed by qPCR of mtDNA genes (ND1, ND5) and nuclear DNA (nDNA) genes (SLCO2B1, SERPINA1). Briefly, the ratios of ΔCt(Ct [ND1]- Ct [SLCO2B1]) or ΔCt(Ct [ND5]-Ct [SERPINA1]) values in the experimental and control groups were calculated as relative mtDNA copy number. The primer sequences are listed in Supplementary Table 2.

### Assay of lysosomal activity

The lysosomal activities of cathepsin B and cathepsin D were determined using Magic Red Cathepsin B (#937, ImmunoChemistry Technologies) and BODIPY FL-Pepstatin A (P12271, Invitrogen), respectively. Fibroblasts were seeded in 96-well plates (black, clear bottom, PerkinElmer) at a density of 5000 cells/well and grown until the cells reached a confluence of 40%. Magic Red Cathepsin B and BODIPY FL-Pepstatin A stock solutions were prepared according to the manufacturer’s instructions. Cells were incubated with Magic Red Cathepsin B (1:25 dilution, 30 min) or BODIPY FL-Pepstatin A (1 µM, 1 h) in the dark at 37℃. Total lysosomal hydrolytic or degradation activity was determined with fluorescein isothiocyanate (FITC)-conjugated 40 K MW dextran (Xian Qiyue Biology, China). Cells were loaded with FITC-dextran (0.5 mg/mL) for 4 h at 37 °C. They were then washed with PBS and chased in fresh culture medium for 20 h to allow the dextran to be transported into the lysosomes or late endosomes. After washing twice with PBS, the cells were incubated for 10 min with Hoechst 33,342 (Immunochemistry Technologies) at a concentration of 1 µg/mL and then washed with PBS prior to imaging. Fibroblasts were imaged using the Opera Phenix High-Content Screening System (PerkinElmer) at 40×objective. Images were analyzed using ImageJ software. The 293T cells loaded with FITC-dextran can also be analyzed by CytoFLEX flow cytometry (Beckman Coulter, USA) without Hoechst 33,342 staining. Quantitative analysis of fluorescence intensities was calculated with FlowJo v10.8.1 software.

### LysoTracker Red (LTR), LysoSensor Green (LSG) and Acridine Orange (AO) staining

Fibroblasts and 293T cells were cultured in 6-well plates and grew to 80% confluence at the end of the experiment. After pharmacological manipulations or resting cultures, the culture medium was removed and adherent cells were detached with 0.25% trypsin-EDTA (CC017, MACGENE) and collected separately in microcentrifuge tubes, followed by centrifugation (300×g, 4 min). Then the cells were resuspended and incubated with fresh medium containing LTR (50 nM, 40739ES50, YEASEN Biology) or LSG (1 µM, 40767ES50, YEASEN Biology) at 37℃ in the dark, mixing gently every 15 min. After washing twice with PBS, the cells were resuspended with PBS and analyzed by flow cytometry using the CytoFLEX flow cytometer (Beckman Coulter, USA). Quantitative analysis of fluorescence intensities was calculated with FlowJo v10.8.1 software.

For AO staining, fibroblasts were seeded at a density of 5000 cells/well in 96-well plates (black, clear bottom, PerkinElmer) and grown to 40% confluence. The culture medium was replaced with fresh medium containing AO (1 µM, Immunochemistry Technologies) and incubated for 30 min at 37℃ in the dark and then washed with PBS. Cells were analyzed using the Opera Phenix High-Content Screening System (PerkinElmer) and ImageJ software. The excitation filter used was 488 nm and the emission filter was 500–530 nm.

### Lysosomal immunoprecipitation (Lyso-IP) proteomics and nucleoside analysis

Lysosomes from cells expressing TMEM192-3xHA were purified according to previously published reports [41]. In brief, cells were seeded in 15 cm dishes at a density suitable to achieve 90% confluence for each sample. Cells were washed twice with ice-cold PBS, scraped in 950 µL ice-cold KPBS buffer (136 mM KCl, 10mM KH2PO4, pH 7.25) and pelleted at 4℃ (2 min, 1000×g). Subsequent steps were performed on ice. The pelleted cells were resuspended in 950 µL of cold KPBS. 25 µL of the resuspended sample was taken for whole cell protein assay and immediately mixed with RIPA lysis buffer containing protease inhibitors. Protein quantification was performed for the whole-cell protein samples to normalize cell numbers for subsequent data analysis. The remaining cells were carefully homogenized with 20 strokes in a tight-fitting Dounce homogenizer. The homogenate was then centrifuged at 1000×g for 2 min at 4 °C to remove nuclei and debris. The supernatant was mixed with 100 µL KPBS prewashed anti-HA magnetic beads and incubated on a rotator at 4℃. For proteomics analysis, the beads were gently shaken with the lysate for 20 min at 6 rpm. For nucleoside or biochemical detection, the beads were shaken at maximum speed (90 rpm) for 3 min. The immunoprecipitates were then gently washed three times with KPBS using a DynaMag Spin Magnet and then eluted in 100 µL KPBS containing 0.5% NP40 (30 min, 4℃) for proteomic analysis or mixed with 50 µL -80℃ frozen methanol: water (80:20, v/v) for subsequent nucleoside analysis. Three biological replicates per group (sgNC vs. sgTYMP) were taken.

Quantitative proteomic analysis was performed by Wuhan GeneCreate Biological Engineering Co., Ltd. A total of 317 proteins or protein fragments were identified. For data quality control, the proteins with 2 missing values in each group were removed, and the remaining missing values were imputed as 1. 260 proteins were then identified for further statistical analysis. Significant differential proteins were defined as proteins with *p* < 0.05 (two-tailed unpaired Welch’s t-test) and log2FC > 0.5 or <-0.5. Volcano plots were calculated using a combination of FC and p-value. Gene Ontology (GO) enrichment analysis, which included cell components (CC) for the downregulated and upregulated proteins, was performed using the clusterProfiler R package.

Nucleoside analysis was performed by Lipidall Technologies Co. Ltd. using ultra-performance liquid chromatography (UPLC) coupled to a quadrupole time-of-flight (Q-TOF) mass spectrometry instrument. Differential analysis of nucleoside metabolites was calculated using semi-quantitative values of the areas under the curve.

### Flow cytometric analysis of JC-1 and MitoSOX staining

Cells were seeded in 6-well plates at a density suitable to achieve 80–90% confluence on the day of collection after treatment with the preparations. After collection by trypsinization, the collected pellets were washed with PBS. According to the manufacturer’s instructions of JC-1 (M8650, Solarbio) or MitoSOX (M36008, Invitrogen), the cells were stained with the prepared JC-1 solution (diluted 1:200 in JC-1 buffer) or MitoSOX solution (diluted 6 µM in medium) and incubated for 20–30 min in the dark at 37℃. During incubation, the cells were gently mixed every 10 min. For JC-1 staining, 5 µM carbonyl cyanide-3-chlorophenylhydrazone (CCCP) was added as a positive control during incubation. Cells were washed, resuspended in PBS and analyzed by CytoFLEX flow cytometry (Beckman Coulter, USA). Quantification was performed using FlowJo v10.8.1 software.

### Cell viability assay

The Cell Titer-Glo Luminescent Cell Viability Assay (Promega) was used for the ATP assay as previously described. In brief, 293T cells (10^4^ per well) were seeded into the black 96-well plates in five replicates. The Cell Titer Glo assay reagent was obtained by mixing the assay buffer and substrate, both of which were brought to room temperature. After 48 h of pharmacological manipulations, 100 µl of the prepared assay reagent was added to each well and mixed by shaking for 2 min. After incubation for 10 min at room temperature, the bioluminescence was read on a Synergy H1 plate reader (BioTek). The amount of ATP present is directly proportional to the intensity of luminescence.

### Flow cytometric analysis of autophagic flux

293T cells expressing mCherry-EGFP-LC3 were seeded in 6-well plates and treated with compounds. After the cells were harvested with trypsin and washed with PBS, they were analyzed by CytoFLEX flow cytometry, recording 10,000 events per experiment. To establish flow cytometry gates, cells treated with 100 µM bafilomycin A1 for 24 h were used as a control for fully inhibited autophagic flux. Starting from the bafilomycin-treated cells, approximately 5% of the cells in the rectangular “autophagy” gate were positive. The changes in autophagy flux were determined as a shift in the cell ratio in this gate after the experimental stimulus. The data were analyzed using FlowJo v10.8.1 software.

### Statistical analysis

Statistical analysis was performed using GraphPad Prism 9, and the Shapiro‒Wilk test was performed to assess the normal distribution. All data were collected and analyzed using a double-blinded approach. Detailed statistical information on the experiments, including the number of replicates (n) and the statistical tests used, are included in the figure legends. Significance levels are indicated as *p < 0.05, **p < 0.01, ***p < 0.001.

## Results

### mtDNA damage and increased mitochondrial content were found in muscle tissue samples from MNGIE patients

**Fig. 1:**
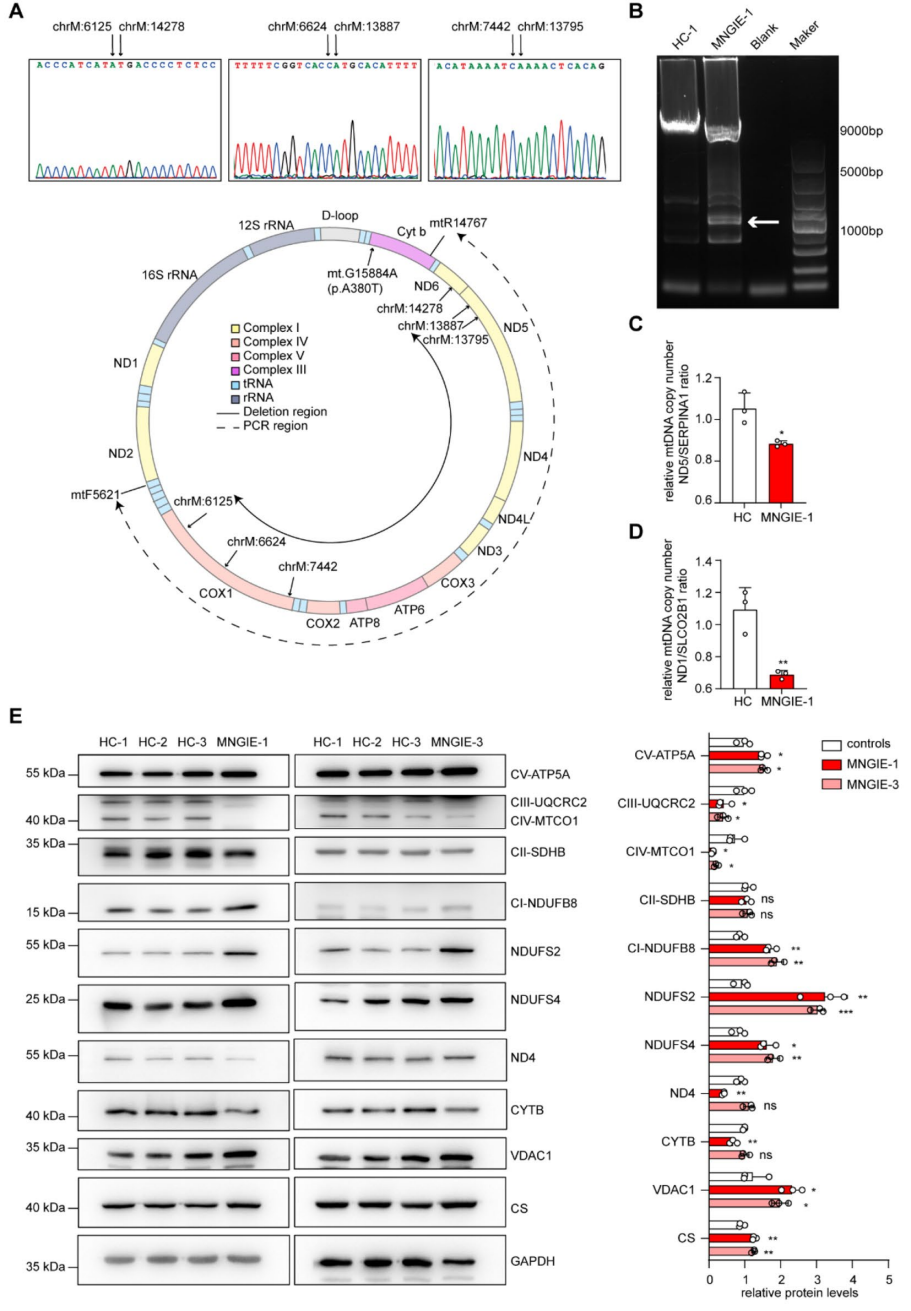
Impaired mtDNA and increased mitochondrial mass in the muscles of MNGIE patients (**A**) Sanger sequencing was used to analyze the detected mtDNA deletions and confirm their specific locations (upper panels). Schematic representation of the region of mtDNA deletions, primer positions and amplification region for long-distance PCR (lower panels). Genes encoded by mtDNA in different complexes are shown in different colors. (**B**) Long-range PCR was used to analyze the length of the deleted mtDNA fragments and validate the deletions. (**C**, **D**) Relative quantification of mtDNA copy number was performed by qPCR. (**E**) Western blot analyzes were performed to evaluate the expression of mitochondrial proteins in muscle tissue from MNGIE patients, with GAPDH serving as an internal control. The corresponding molecular weight markers (kDa) are shown on the left. An unpaired two-tailed Student’s t-test was used to calculate *p*-values. Three independent experiments were performed to obtain the mean ± standard deviation (* *p* < 0.05, ** *p* < 0.01, *** *p* < 0.001)

mtDNA impairment has long been recognized as a characteristic feature of MNGIE. By mtDNA sequencing of muscle tissue from one of the three MNGIE patients recruited by our center (MNGIE-1), we also identified three large mtDNA deletions (chrM:6625–13,886, chrM:6126–14,277, chrM:7443–13,794) (Fig. 1A) and several mtDNA point mutations (Supplementary Table 3) in the muscle tissue of the MNGIE-1 patient. To validate the deletions, we performed long-distance PCR, which showed the presence of approximately 8 kb deletions in a specific proportion (Fig. 1B). In addition, a reduced mtDNA copy number was detected in the muscle sample of patient MNGIE-1 (Fig. 1C, D). These findings indicate that both the integrity and quantity of mtDNA sequences are impaired in the muscle sample of our MNGIE patient.

Western blot analysis of muscle tissue from MNGIE-1 and MNGIE-3 patients revealed a significant decrease in the expression of mitochondrial respiratory chain complex III and complex IV subunits, suggesting the presence of defects in the mitochondrial respiratory chain (Fig. 1E). In addition, we found decreased expression of MT-ND4 and MT-CYTB proteins, the latter possibly related to the *CYTB* missense mutation detected in the muscle sample of MNGIE-1 (Fig. 1E, Supplementary Table 3). However, the expression of many nuclear-encoded subunits of complex I and complex V (NDUFB8, NDUFS4, NDUFS2 and ATP5A) was increased in the muscle tissue of MNGIE patients compared to the control group (Fig. 1E). We also found increased levels of the mitochondrial reference protein VDAC1 and citrate synthase (CS) in the muscle tissue of MNGIE patients. However, this phenomenon was not observed in the muscle tissue of patients with the m.3243 A > G mutation (Fig. S1A). These data suggest that not only mtDNA and mitochondrial respiratory chain are impaired in muscle tissue of MNGIE patients in this study, but also mitochondrial content is increased.

### *TYMP* deficiency leads to lysosomal dysfunction and impairs mitochondrial clearance

**Fig. 2:**
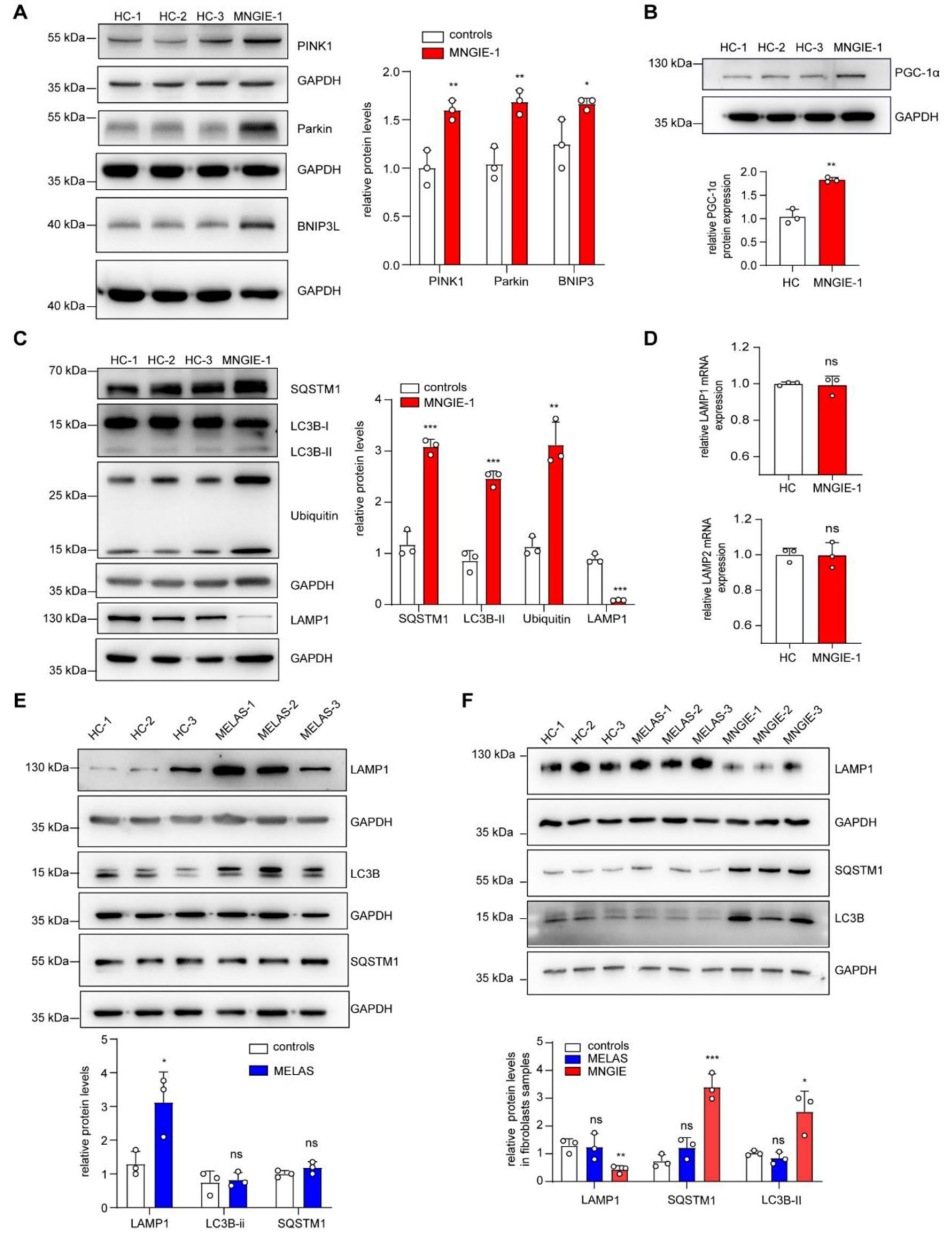
Lysosomal dysfunction involved in impaired mitochondrial clearance of MNGIE patients (**A**-**C**) Western blot analysis was performed to evaluate the expression of mitochondrial autophagy (**A**), mitochondrial biogenesis (**B**) and autophagosome and lysosome proteins (**C**) in muscle tissue of MNGIE patients, using GAPDH as an internal reference. The corresponding molecular weight markers (kDa) are indicated on the left. (**D**) qPCR analysis was used to determine the levels of LAMP1 and LAMP2 mRNA in muscle tissue of MNGIE patients. (**E**) Western blot analysis of autophagosome and lysosomal protein expression in muscle tissue from m.3243 A > G MELAS patients, using GAPDH as an internal reference. (**F**) Expression of lysosomal and autophagosome proteins in skin fibroblasts from MNGIE and m.3243 A > G MELAS patients. Data are presented as mean ± standard deviation and are pooled from three independent experiments. Statistical significance was determined using unpaired two-tailed Student’s t-test: *p < 0.05, **p < 0.01, ***p < 0.001

To investigate the reasons for the abnormal increase in mitochondrial content, the proteins related to mitochondrial clearance and biogenesis were analyzed in the muscle of MNGIE patients by Western blot. As shown in Fig. 2A, the protein levels of Parkin, PINK1 and BNIP3L were increased in the muscle tissue of MNGIE patients, indicating the activation of mitochondrial autophagy to clear damaged mitochondria with mutated mtDNA. In addition, the expression of PGC-1α was increased (Fig. 2B), indicating coordinated activation of mitochondrial biogenesis pathways. The completion of mitophagy involves the fusion of mitochondria-containing autophagosomes with lysosomes, followed by the degradation of mitochondria in lysosomes. To further investigate whether autophagosomes were efficiently degraded or whether there was disruption in autophagic flux, the protein expression levels of autophagy markers (including LC3B, SQSTM1 and ubiquitin) and the lysosome marker protein LAMP1 were analyzed by Western blot in muscle samples from MNGIE patients. As shown in Fig. 2C and Fig. S2A-C, the levels of LC3B-II, SQSTM1 and ubiquitin were increased, while LAMP1 protein level was significantly decreased in the muscle of MNGIE patients, indicating impaired lysosomal function and blocked autophagic flux. In addition, mRNA levels of LAMP1 and LAMP2 in muscles from MNGIE patients showed no significant changes (Fig. 2D), suggesting that transcriptional activation of lysosome biogenesis was not appreciably impaired. These data suggest that the combination of activated mitochondrial autophagy, enhanced mitochondrial biogenesis, lysosomal defects and impaired autophagic flux contributes to the abnormal increase in mitochondrial content. It is worth noting that the levels of PINK1 and BNIP3L were decreased in the muscle of m.3243 A > G MELAS patients compared with the control group, indicating inhibition of mitochondrial autophagy initiation in MELAS (Fig. S1B). In the muscles of m.3243 A > G MELAS patients, protein expression of LAMP1 was increased rather than decreased. No significant changes were detected in autophagy-related proteins such as LC3B-II and SQSTM1 (Fig. 2E). These results suggest that the inhibition of mitochondrial autophagy in m.3243 A > G MELAS occurs in the early recruitment phase, accompanied by changes in lysosomal homeostasis. However, this does not lead to the lysosomal dysfunction observed in MNGIE patients.

As an easily accessible cell carrying the genotype information of patients, fibroblast is commonly used as a cell model in mitochondrial disorders and have also been employed in research on nucleoside metabolism and MNGIE pathogenesis [42–44]. To clarify the changes in lysosomal function in MNGIE patients, fibroblasts from patient samples were further investigated. Western blot analysis revealed a significant decrease in LAMP1 protein expression in skin fibroblasts from MNGIE patients compared to the control group (Fig. 2F), which is consistent with the reduced LAMP1 expression in muscle samples from MNGIE patients. In addition, increased levels of SQSTM1 and LC3B-II proteins were found in MNGIE fibroblasts. In contrast, there were no significant changes in LAMP1, SQSTM1 and LC3B-II protein levels in skin fibroblasts from m.3243 A > G MELAS patients. These data further suggest that the lysosomal dysfunction observed in MNGIE patients may not be directly caused by mitochondrial damage, but that the unique lysosomal impairment in MNGIE indicates additional effects of TP defects on lysosomes.

### TP function deficiency leads to alkalization of the lysosomes and reduced activity of lysosomal enzymes

**Fig. 3:**
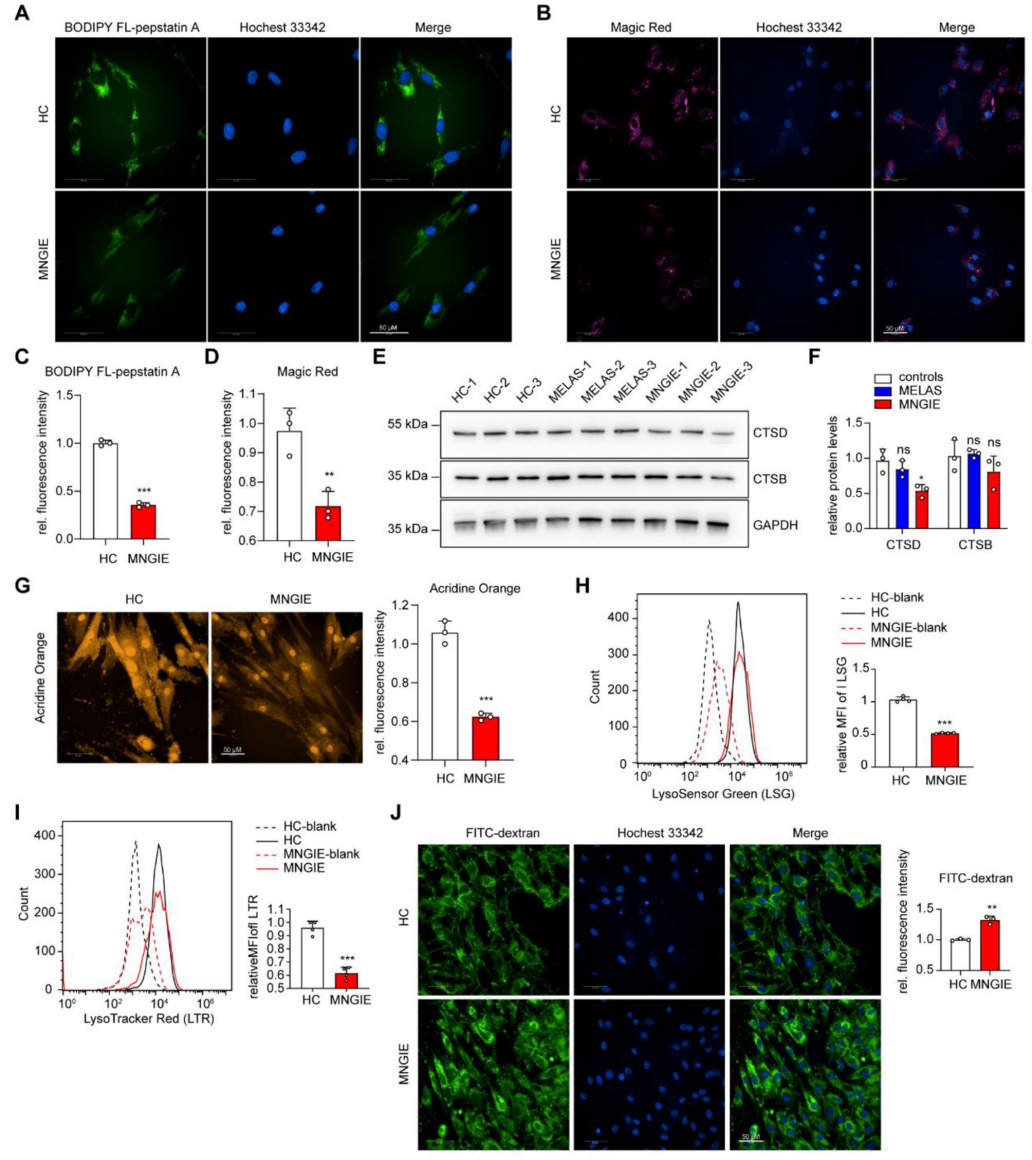
*TYMP* deficiency leads to a reduction in lysosomal activity and acidity (**A**) Fluorescence imaging shows a decrease in fluorescence intensity of BODIPY FL-Pepstatin A in MNGIE fibroblasts compared to control cells. (**B**) Fluorescence imaging shows a decrease in the fluorescence intensity of Magic Red in MNGIE fibroblasts compared to control cells. (**C**) Quantitative analysis of the relative fluorescence intensity of BODIPY FL-Pepstatin A. (**D**) Quantitative analysis of the relative fluorescence intensity of Magic Red. (**E**) Western blot analysis of CTSD and CTSB protein expression in skin fibroblasts from MNGIE and m.3243 A > G MELAS patients using GAPDH as a loading control. (**F**) Relative quantification analysis of CTSD and CTSB protein expression. (**G**) Fluorescence imaging and relative quantitative analysis showed a decrease in fluorescence intensity of acridine orange in MNGIE fibroblasts compared to control cells. (**H**) Flow cytometric detection and quantitative analysis showed a decrease in the fluorescence intensity of LSG in MNGIE fibroblasts compared to control cells. (**I**) Flow cytometric detection and quantification analysis showed a decrease in the fluorescence intensity of LTR in MNGIE fibroblasts compared to control cells. (**J**) Fluorescence imaging after FITC-dextran treatment showed an increase in the fluorescence intensity of FITC in MNGIE fibroblasts compared to control cells. Unpaired two-tailed Student’s t-test was used to calculate the *p*-values. Data are given as mean ± standard deviation (* *p* < 0.05, ** *p* < 0.01, *** *p* < 0.001)

In addition to the lysosomal membrane proteins, the hydrolytic enzymes within the lysosomes are also important functional components. To analyze the protease activity within the lysosomes, we performed fluorescence assays with Magic Red and BODIPY FL-Pepstatin A to detect the enzymatic activity of cathepsin B (CTSB) and cathepsin D (CTSD), respectively. Surprisingly, both Magic Red and BODIPY fluorescence intensities were significantly decreased in MNGIE fibroblasts, indicating a reduction in active CTSB and CTSD proteases due to *TYMP* deficiency (Fig. 3A-D). Additionally, a slight downregulation of CTSD protein levels was observed in MNGIE fibroblasts, without significant changes in CTSB levels (Fig. 3E, F). Lysosomal hydrolytic enzymes are all acidic hydrolases with an optimal pH range of 3.5–5.5 and pH-dependent protein maturation properties [45]. Their enzymatic activity and protein stability are very sensitive to changes in lysosomal pH. Therefore, the decreased activity of CTSB and CTSD proteins indirectly indicates an upward shift in lysosomal pH in MNGIE fibroblasts. To further analyze the lysosomal acidity in MNGIE fibroblasts, we stained the cells with AO, LSG and LTR. As shown in Fig. 3G-I, the fluorescence intensities of AO, LSG and LTR were decreased compared to control fibroblasts, indicating impaired acidic conditions in the lysosomal lumen. Taken together, the decreased levels of LAMP1 protein, decreased activity of lysosomal hydrolytic enzymes, and impaired acidic environment in MNGIE fibroblasts suggest that the deficiency of TP leads to disruption of lysosomal membrane integrity and a decrease in the number of functional lysosomes. In addition, we further investigated the degradation capacity of lysosomes in MNGIE fibroblasts by treating them with FITC-dextran and measuring the fluorescence intensity. The results showed a significant increase in FITC fluorescence intensity in MNGIE fibroblasts compared to control cells (Fig. 3J), further reflecting the impairment of lysosomal degradation capacity. To investigate the effect of mitochondrial damage on lysosomal dysfunction in MNGIE fibroblasts, we also performed Magic Red and acridine orange fluorescence analysis in m.3243 A > G MELAS fibroblasts. The results showed no significant changes in fluorescence intensity compared to control cells (Fig. S3A-D), suggesting that TP function deficiency acts directly on lysosomal damage rather than indirectly via abnormal mitochondrial function.

### Both *TYMP* gene knockout and chemical inhibition can lead to mitochondrial dysfunction and lysosomal damage

**Fig. 4:**
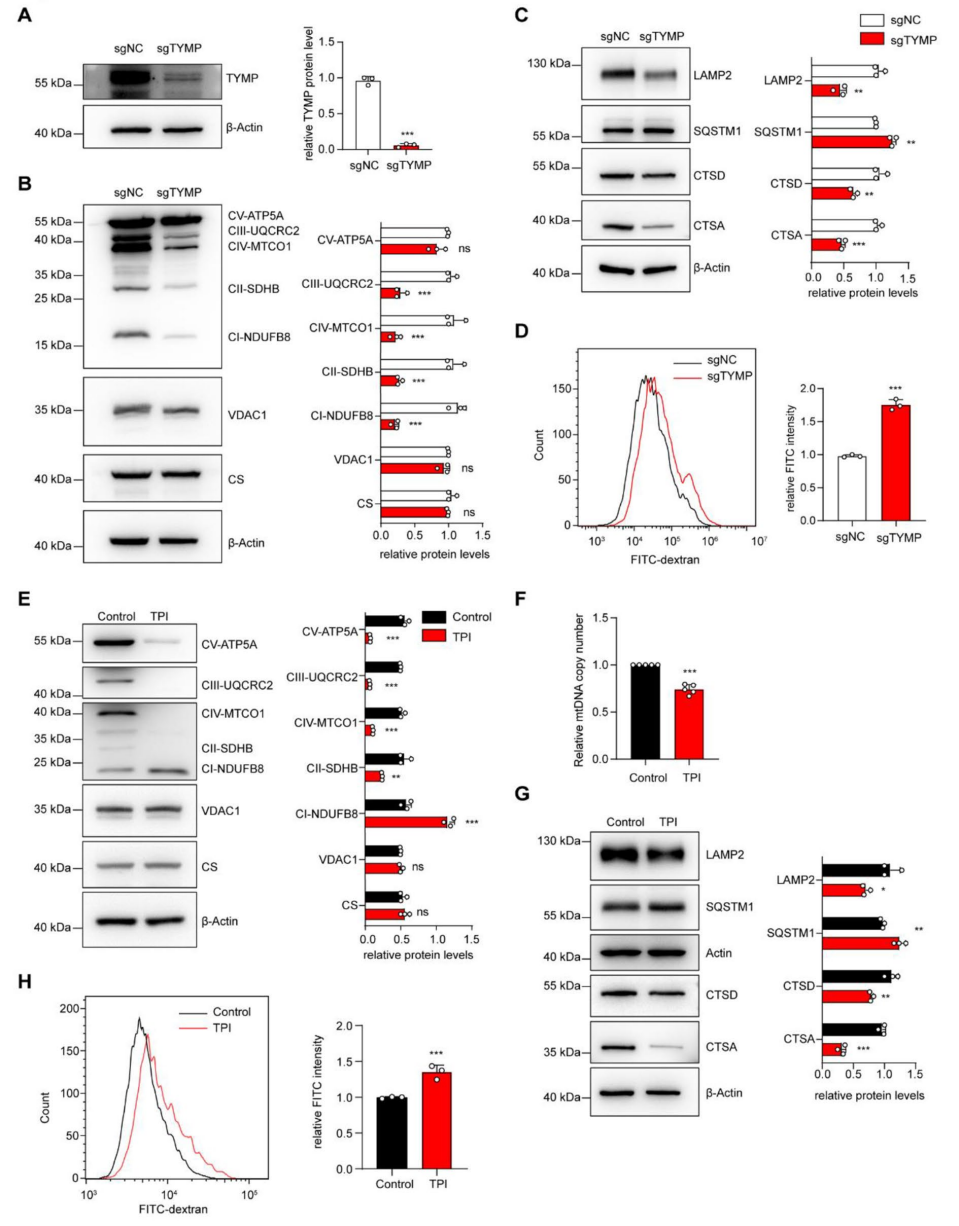
Genetic deletion and chemical inhibition of TP cause mitochondrial and lysosomal damage (**A**) CRISPR/Cas9-mediated gene knockout of *TYMP* resulted in almost complete loss of TP protein expression in 293T cells (approx. 95% loss). (**B**) Western blot detection and relative quantification analysis of mitochondrial protein expression in 293T cells with *TYMP* knockout. (**C**) Western blot detection and relative quantification analysis of lysosomal and autophagic protein expression in *TYMP* knockout 293T cells, using β-actin as a loading control. (**D**) Flow cytometric detection and quantification analysis showed an increase in the relative fluorescence intensity of FITC-dextran in *TYMP* knockout cells compared to control cells. (**E**-**G**) After chemical inhibition of TP enzyme activity in 293T cells (48-hour treatment with 2 µM tipiracil), Western blot detection and relative quantification analysis of mitochondrial (**E**), lysosomal and autophagic protein expression (**G**) were performed in TP enzyme-inhibited cells. (**F**) qPCR analysis of mtDNA copy number showed a decrease in mtDNA copy number in cells after TP enzyme inhibition. (**H**) Flow cytometric detection and quantification analysis showed an increase in the relative fluorescence intensity of FITC-dextran in cells after TP enzyme inhibition with tipiracil compared to control cells. An unpaired two-tailed Student’s t-test was used to calculate *p*-values. Data are expressed as mean ± standard deviation (* *p* < 0.05, ** *p* < 0.01, *** *p* < 0.001)

To further investigate the effects of *TYMP* deficiency on mitochondrial and lysosomal homeostasis, we used gene knockout and chemical inhibition in 293T cells to reduce *TYMP* expression and TP activity, respectively. *TYMP* was knocked out in 293T cells using CRISPR-Cas9 and the knockout efficiency was confirmed by Western blot analysis (Fig. 4A). Further Western blot analysis of mitochondrial respiratory chain proteins in *TYMP*-KO 293T cells showed a decrease in protein levels of respiratory chain complex subunits I, II, III and IV compared to control, indicating impaired mitochondrial respiratory chain function (Fig. 4B). However, the expression of VDAC1 and CS showed no significant increase in *TYMP*-KO 293T cells, suggesting that the mitochondrial content did not change significantly. This is inconsistent with the observed mitochondrial accumulation in muscle tissue of MNGIE patients, which may be related to the dilution effects of rapid cell division and varied half-lives of mitochondrial proteins in different tissues [46, 47]. In addition, we analyzed the expression of lysosomal proteins and SQSTM1 protein which is a marker for autophagosomes and usually used to evaluate the autophagic degradation [48]. We found a decrease in LAMP2, CTSD and CTSA proteins and an increase in SQSTM1 protein expression, which is consistent with the results in fibroblasts from MNGIE patients (Fig. 4C). We then treated *TYMP*-KO 293T cells with FITC-dextran and performed flow cytometry analysis, which showed an increase in FITC intensity compared to control 293T cells, indicating impaired lysosomal degradation function (Fig. 4D).

Consistent with the data from *TYMP* gene knockout in 293T cells, chemical inhibition of TP activity with the TP protein inhibitor tipiracil also resulted in impaired expression of subunits of the mitochondrial respiratory chain complex (Fig. 4E). Interestingly, inhibition of TP activity led not only to a decrease in several respiratory chain proteins, but also to an increase in complex I subunit NDUFB8 proteins, which is consistent with the respiratory chain protein profile observed in muscle tissue from MNGIE patients. The mtDNA copy number also decreased after inhibition of TP activity (Fig. 4F). Furthermore, inhibition of TP activity resulted in significant lysosomal damage in the cells, including decreased levels of LAMP2, CTSA and CTSD proteins, increased levels of SQSTM1 and accumulation of FITC dextran substrate (Fig. 4G, H). These results indicate that both *TYMP* gene knockout and chemical inhibition can induce mitochondrial dysfunction and lysosomal damage, suggesting that impairment of TP activity is sufficient to disrupt mitochondrial and lysosome homeostasis.

### Characterization of lysosomal protein features in *TYMP*-deficient 293T cells

**Fig. 5:**
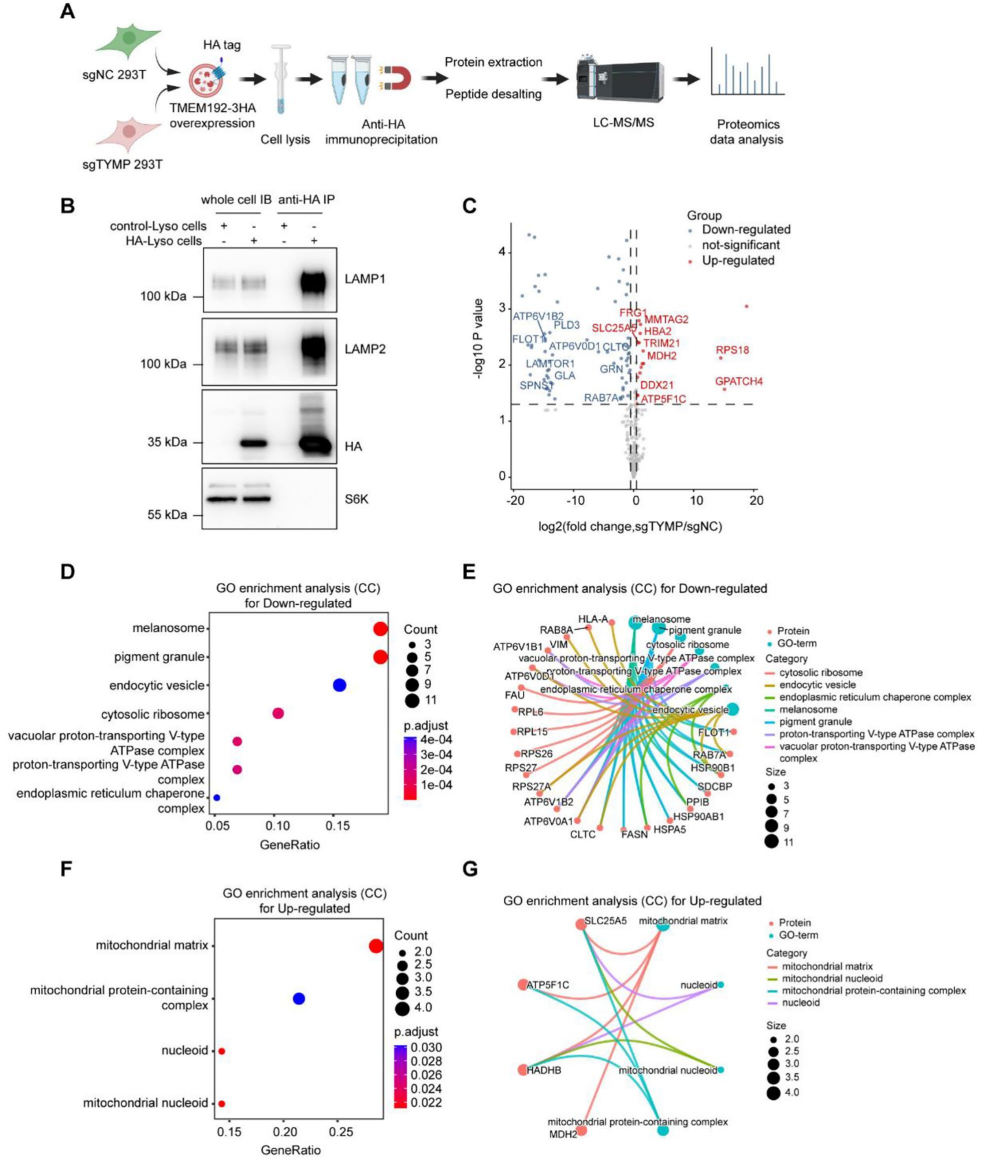
Characterization of lysosomal protein changes in 293T cells with *TYMP* deficiency (**A**) Schematic diagram illustrating the process. Starting from the stable cell line overexpressing TMEM192-3×HA, *TYMP* gene knockout or control cells were prepared. Using the Lyso-IP technique, lysosomes were isolated from cells with or without *TYMP* gene knockout and extracted to perform further proteomic analysis. (**B**) Western blot analysis of whole cell samples from stable transfected cell lines overexpressing TMEM192-3×HA and control cells and lysosome samples after Lyso-IP separation: Overexpression of TMEM192-3×HA in cells had no effect on basal expression of lysosomal proteins and cytoplasmic protein S6K. Enrichment of lysosomal proteins by anti-HA magnetic beads is effective and not contaminated with cytoplasmic proteins. (**C**) Volcano plot showing different proteins between lysosomes extracted from *TYMP* knockout cells and control cells (*p* < 0.05, *logFC* > 0.5). (**D-G**) GO enrichment analysis (cellular components) of upregulated (**D, E**) and downregulated (**F, G**) proteins in lysosomes after *TYMP* knockout, represented by a bubble plot (**D, F**) and a gene set protein network diagram (**E, G**)

To investigate the specific changes in lysosomal proteins caused by *TYMP* deficiency, we used the Lyso-IP technique to isolate lysosomes from wild-type 293T cells and *TYMP* gene knockout 293T cells, and then performed label-free proteomic analysis (Fig. 5A). As shown in Fig. 5B, the lysosomal markers LAMP1 and LAMP2 were enriched in the HA-labelled samples, which did not contain cytoplasmic proteins such as S6K, confirming the high-purity isolation of lysosomes by the Lyso-IP method.

Proteomic analysis of the Lyso-IP results revealed significant differences in lysosomal protein composition between lysosomes isolated from *TYMP* knockout cells and wild-type cells (Fig. 5C). Further differential protein GO enrichment analysis revealed an increase in mitochondrial protein content in lysosomes from *TYMP* knockout cells, including mitochondrial matrix, respiratory chain complexes and nucleoid proteins, while lysosomal protein components were reduced, including vesicles, endosomes and V-type ATPase complex proteins (Fig. 5D-G). Damaged or senescent mitochondria are normally degraded by lysosomes as substrates. However, based on our proteomic data from isolated lysosomes, an increase in mitochondrial protein components was detected in lysosomes from *TYMP*-deficient cells, which may indicate impaired degradation of mitochondrial substrate proteins and other substrates by lysosomes. Considering the proteomic data of purified lysosomal proteins and the results of lysosomal function experiments performed in *TYMP*-deficient cells, these results collectively suggest that *TYMP* deficiency leads to lysosomal dysfunction characterized by increased substrate concentrations, impaired acidification, impaired limiting membrane integrity, and impaired vesicle fusion.

### *TYMP* deficiency leads to an accumulation of nucleosides in the lysosomes and impairs lysosomal function and mitochondrial homeostasis

**Fig. 6:**
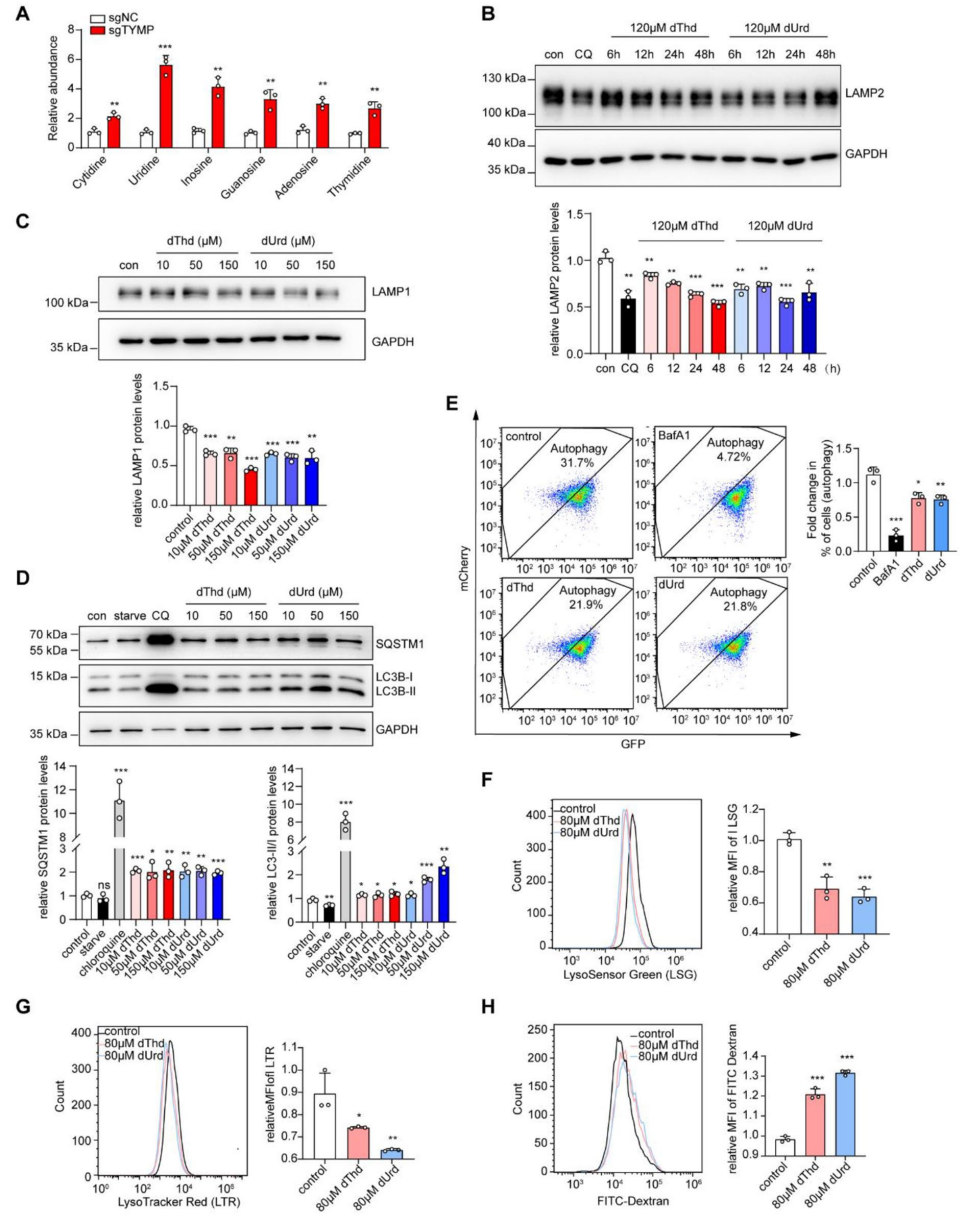
Lysosomal accumulation of nucleosides caused by *TYMP* deficiency impairs lysosomal function (**A**) After lysosome isolation with Lyso-IP, mass spectrometric analysis of nucleoside compounds in lysosomes of *TYMP* knockout cells and control cells. (**B**) Western blot detection and relative quantification of the expression of the lysosomal protein LAMP2 in 293T cells treated with chloroquine (positive control, 100 µM, 24 h) and different durations (6, 12, 24, 48 h) with 120 µM thymidine or deoxyuridine. GAPDH was used as internal reference. (**C**) Western blot detection and relative quantification of the expression of the lysosomal protein LAMP1 in 293T cells treated with different concentrations (10, 50, 150 µM) of thymidine or deoxyuridine for 24 h. (**D**) Western blot detection and relative quantification of the expression of autophagy proteins LC3B and SQSTM1 in 293T cells treated with 24 h starvation (negative control, Earle’s balanced salt solution, EBSS), chloroquine (positive control, 100 µM, 24 h) and different concentrations (10, 50, 150 µM) of thymidine or deoxyuridine. (**E**) Flow cytometric analysis and relative quantification of mCherry-LC3B-GFP stably expressing 293T cells treated with bafilomycin A1 (positive control, 100 µM, 24 h) and 80 µM thymidine or deoxyuridine for 24 h. Fluorescence changes after inhibition by bafilomycin A1 were used to define the flow-through analysis window for the assessment of “autophagy activity”. (**F-H**) Flow cytometric detection and relative quantification of the fluorescence intensities of LSG (**F**), LTR (**G**) and FITC-dextran (**H**) in 293T cells treated with 80 µM thymidine or deoxyuridine for 24 h. An unpaired two-tailed Student’s t-test was used to calculate *p*-values. Data are expressed as mean ± standard deviation (* *p* < 0.05, ** *p* < 0.01, *** *p* < 0.001)

To further explore and analyze the underlying mechanisms of lysosomal dysfunction caused by *TYMP* deficiency, we considered lysosomes as organelles responsible for the degradation of nucleic acids to nucleosides, which are then transported back to the cytoplasm by transporter proteins. Therefore, we hypothesized that the accumulation of thymidine and deoxyuridine in the cytoplasm of *TYMP*-deficient cells might limit the normal export of nucleosides from lysosomes. Nucleosides, which are weakly basic substances, may furthermore compete for available protons in the lysosomal lumen, leading to lysosomal alkalinization, functional defects and increased membrane permeability. To demonstrate this, we performed Lyso-IP to isolate lysosomes and further analyzed the nucleoside content in *TYMP* knockout and control cells (293T). The results showed a significant increase in various nucleosides extracted from the lysosomes of sgTYMP-293T cells compared with the control, including adenosine, thymidine, cytidine, uridine, inosine, and guanosine, possibly inducing lysosomal alkalinization (Fig. 6A). These results are consistent with our hypothesis and suggest that the accumulation of nucleosides in the lysosomal lumen may be the cause of lysosomal defects in *TYMP* deficiency.

To further investigate the effects of excessive thymidine and deoxyuridine on lysosomal function, we treated 293T cells with high concentrations of these nucleosides and performed experiments to assess the autophagy pathway and lysosomal acidity. After thymidine and deoxyuridine treatment, the level of various nucleosides showed increased in lysosome (Fig. S4A). As shown in Fig. 6B-D, after treatment with increasing concentrations and time gradients of thymidine and deoxyuridine (with EBSS starvation and chloroquine treatment as negative and positive control respectively), the expression of LAMP2 and LAMP1 proteins was reduced, while the levels of LC3B and SQSTM1 were increased, resembling the phenotype of *TYMP*-KO 293T cells without an apparent dosage and time-dependent effect. In addition, we treated stable mCherry-LC3B-GFP-expressing 293T cells with thymidine and deoxyuridine (with bafilomycin A1 treatment as positive control) and analyzed them by flow cytometry. The results showed a decrease in the percentage of cells with completed degradation of autophagosomes compared to the control after high-dose thymidine and deoxyuridine treatment (Fig. 6E). We also stained thymidine- and deoxyuridine-treated 293T cells with LSG and LTR. Flow cytometric analysis showed a decrease in LSG and LTR fluorescence intensity after treatment with thymidine and deoxyuridine (Fig. 6F, G). In addition, FITC-dextran flow cytometry analysis showed a significant decrease in the degradation of FITC-dextran in the high thymidine and deoxyuridine dose group compared to the control group (Fig. 6H). These results suggest that the accumulation of thymidine and deoxyuridine in the cells may cause lysosomal alkalinization and impair lysosomal degradation capacity.

We then treated the cells with thymidine and deoxyuridine and performed JC-1 staining to determine the mitochondrial membrane potential. The results showed a decrease in mitochondrial membrane potential in the cells after treatment with thymidine and deoxyuridine, indicating increased mitochondrial damage (Fig. S5A). In addition, measurements of cellular ATP levels, mitochondrial DNA copy number and mitochondrial superoxide production showed a decrease in ATP levels, a decrease in mitochondrial copy number and an increase in mitochondrial reactive oxygen species (ROS) production after thymidine and deoxyuridine treatment (Fig. S5B-D). Taken together, these results suggest that the accumulation of thymidine and deoxyuridine in cells can disrupt mtDNA homeostasis, leading to mitochondrial dysfunction, as well as cause nucleoside accumulation in lysosomes, leading to lysosomal defects, which in turn impairs mitochondrial clearance and exacerbates mitochondrial dysfunction.

## Discussion

Lysosomes play a crucial role in mitochondrial quality control as the destination of mitochondrial clearance and are closely associated with mitochondrial function [21, 49, 50]. In this study, we used various methods such as tissue extraction, primary cell culture, gene knockout, and chemical inhibition to reveal lysosomal dysfunction in MNGIE disease or TP deficiency. In addition, we isolated and analyzed lysosomes from *TYMP*-deficient cells using Lyso-IP and proteomic techniques, investigated changes in lysosomal proteins, and confirmed the accumulation of nucleosides in lysosomes. In addition, we validated changes in lysosomal dysfunction and mitochondrial homeostasis caused by excessive accumulation of thymidine and deoxyuridine in cells.

Compared to previously discovered lysosomal damage caused by mitochondrial dysfunction, this study showed a distinctive form of lysosomal dysfunction in MNGIE. Previous studies have shown that knockout or knockdown of certain mitochondrial proteins such as AIF, OPA1, PINK1 and UQCRC1 not only leads to mitochondrial dysfunction, but also impairs lysosomes, resulting in enlarged lysosomes, the appearance of large vacuoles and impaired lysosomal enzymatic activity. However, the protein content of LAMP1 remained normal [51, 52]. This is consistent with our findings of increased LAMP1 expression in muscle samples from m.3243 A > G MELAS patients. In addition, knockout of the p32 gene leading to mitochondrial translation defects was found to result in an increased number of lipofuscin-containing lysosomes in mouse hearts, indicating impaired lysosomal and autophagic functions [53]. In addition, a recent study discovered an increased number of lysosomes and altered spatial localization in ragged red fibers (RRF) of a mitochondrial myopathy mouse model, suggesting abnormal lysosomal homeostasis in RRF [54]. Since muscle samples from m.3243 A > G MELAS patients contain a large amount of RRF, the increased level of LAMP1 observed in the muscles of MELAS patients may be related to the increased number of lysosomes in RRF. Finally, an acute increase in ROS concentration may also cause permeabilization of lysosomal membranes and a decrease in LAMP1 protein levels, leading to cell death [55–57]. Collectively, lysosomal damage caused by mitochondrial dysfunction or ROS-induced damage can be categorized into two situations: lysosomal enlargement and increased lysosomal quantity due to long-term mitochondrial defects or lysosomal membrane permeabilization and cell death due to acute ROS production. However, in MNGIE patients or TP-deficient cells, lysosomes do not show increased volume despite long-term abnormalities of mitochondrial proteins, but rather reduced or dysregulated expression of lysosomal membrane proteins. This abnormality of the lysosomal membrane differs from the permeabilization of the lysosomal membrane and does not lead to a significant tendency to cell death. We therefore conclude that TP deficiency leads to lysosomal dysfunction characterized by membrane dysregulation without marked cell death.

TP is a key enzyme in nucleoside metabolism, and its defects can lead to the accumulation of large amounts of thymidine and deoxyuridine in cells, which is also the underlying cause of the mitochondrial dysfunction observed in MNGIE [2]. Nucleosides are the end products of lysosomal degradation of various forms of nucleic acids in cells and are recycled to the cytoplasm via carrier proteins by facilitated diffusion along concentration gradients [38, 39, 58, 59]. Previous studies have revealed that the ENT3 protein in mammalian cells is a lysosome-localized nucleoside transporter with pH-dependent transport activity that is responsible for the transport of various nucleosides such as adenosine, uridine and cytidine from the lysosomal lumen into the cytoplasm [39, 60]. Therefore, we speculate that the long-term accumulation of thymidine and deoxyuridine in the cytoplasm of *TYMP*-deficient cells may hinder lysosomal nucleoside recycling, leading to increased nucleoside accumulation in the lysosomal lumen. In addition, nucleosides have weak alkaline properties and can bind hydrogen protons, thereby alkalizing lysosomes. Disruption of the acidic environment of lysosomes reduces lysosomal enzyme activity, impairs normal substrate hydrolysis and leads to a range of lysosomal dysfunctions. Consistent with our hypothesis, we found increased levels of various nucleosides such as cytidine nucleoside, uridine, guanosine nucleoside, adenosine and cytidine in lysosomes extracted from *TYMP*-deficient cells, as well as increased levels of mitochondrial proteins and decreased levels of lysosomal membrane V-ATPase proteins in lysosomal protein extracts from *TYMP*-deficient cells. Similar to mitochondrial dysfunction caused by mitochondrial overload with dThd and dUrd in MNGIE, these results suggest that the accumulation of lysosomal nucleosides is the cause of the imbalance of lysosomal membrane proteins, disruption of the acidic environment, and impairment of degradation capacity. In addition, many antiretroviral drugs such as zidovudine, didanosine and stavudine, which are nucleoside analogs, have also been found to inhibit autophagy, disrupt the acidic environment in the lysosomes, increase mitochondrial mass and increase the production of reactive oxygen species [61–63]. These nucleoside analogs can also be transported by ENT3, which is consistent with our results and suggests a possible mechanism of inhibition of lysosomal nucleoside recycling by nucleoside analogs [64]. Interestingly, Stankov et al. found that the cytidine analogs zidovudine and stavudine can inhibit adipocyte proliferation and lipid synthesis, which is consistent with the inhibition of fatty acid synthesis metabolism in *TYMP* deficiency observed in our previous study and further highlights the importance of TP protein in cell metabolism homeostasis and organelle function [40, 61, 62].

Our results suggested that in the context of MNGIE, mitochondrial and lysosomal damage coexist and reinforce each other: on the one hand, defects in mitochondrial energy metabolism increase the generation of ROS, which may in turn increase the permeability of lysosomal membranes; on the other hand, lysosomal dysfunction impedes the normal clearance of mitochondria, leading to the persistent presence of abnormal mitochondria with mtDNA defects. Similarly, defects in the ENT3 protein have been found to simultaneously lead to mitochondrial damage and lysosomal dysfunction, resulting in an accumulation of mitochondrial redundancy, increased ROS and increased lysosomal volume [65, 66]. The mechanism behind this phenomenon may be consistent with the mechanism of mitochondrial and lysosomal nucleoside accumulation caused by TP deficiency, as studies have shown that ENT3 is localized not only in lysosomes but also in mitochondria [60]. Interestingly, two key amino acids responsible for pH sensing, glutamic acid at position 447 and aspartic acid at position 219, have opposite orientations in lysosomes and mitochondria. ENT3 localized in lysosomes transports nucleosides from lysosomes into the cytoplasm, whereas ENT3 localized in mitochondria is responsible for the transport of nucleosides from the mitochondrial intermembrane space into the mitochondrial matrix. Therefore, ENT3 defects not only lead to lysosomal nucleoside accumulation, but also impair nucleoside availability in the mitochondria. Based on this feature of ENT3, we propose a pathogenic model for MNGIE: cytoplasmic accumulation of thymidine and deoxyuridine caused by TP deficiency enters mitochondria on the one hand, leading to imbalance of mitochondrial nucleoside levels and disruption of mtDNA homeostasis, and on the other hand, it causes excessive accumulation of nucleosides in lysosomes, resulting in lysosomal dysfunction.

Although our study provides theoretical insights, it is important to recognize its limitations. Due to the complex interplay between mitochondria and lysosomes, we compared the phenotypes of tissues and cells from MNGIE patients with those from m.3243 A > G MELAS patients to clarify the direct effects of TP deficiency on lysosomal dysfunction and exclude the involvement of mitochondrial damage. However, this approach cannot completely exclude the possibility that mitochondrial damage due to mtDNA defects contributes to lysosomal dysfunction in the TP deficiency model. Nonetheless, the lysosomal damage observed in TP-deficient cells differs from the features resulting from mitochondrial dysfunction observed in previous studies, suggesting in part a direct influence of TP deficiency on lysosomal damage. Future studies should investigate the effects of TP deficiency on lysosomal function by fully correcting mtDNA defects in TP-deficient cells using techniques such as mtDNA replacement. Besides, while the 293T cell model allowed us to investigate certain aspects of TP deficiency and lysosomal malfunction, it may not fully recapitulate the mitochondria accumulation seen in MNGIE muscles. A more comprehensive evaluation of the disturbed mitochondrial and lysosomal homeostasis using in vivo models or organoids derived from patients is worthy in the future.

## Conclusion

Therefore, our study provided evidence that TP deficiency leads to nucleosides overload in lysosomes and lysosomal dysfunction, revealing the widespread disruption of organelles underlying MNGIE. This contributes to understanding the pathophysiological mechanism of MNGIE as an inherited metabolic disease rather than just a mitochondrial disease.

### Electronic supplementary material

Below is the link to the electronic supplementary material.

Supplementary Material 1

## Data Availability

The data that support the findings of this study are available from the corresponding author upon reasonable request.

## Abbreviations

AO: Acridine Orange
ATCC: American Type Culture Collection
CC: Cell Components
CCCP: Carbonyl Cyanide-3-Chlorophenylhydrazone
DMEM: Dulbecco’s Modified Eagle Medium
dThd: Thymidine
dUrd: Deoxyuridine
EBSS: Earle’s balanced salt solution
FITC: Fluorescein Isothiocyanate
GO: Gene Ontology
HC: Healthy Control
KPBS: Potassium Phosphate Buffered Saline
LSG: LysoSensor Green
LTR: LysoTracker Red
Lyso-IP: Lysosome Immunoprecipitation
MELAS: Mitochondrial Encephalopathy, Lactic Acidosis and Stroke-like Episodes
MNGIE: Mitochondrial Neurogastrointestinal Encephalomyopathy
mtDNA: mitochondrial DNA
qPCR: quantitative PCR
Q-TOF: Quadrupole Time-of-Flight
ROS: Reactive Oxygen Species
RRF: Ragged Red Fiber
TBST: Tris-Buffered Saline with Tween 20
TP: Thymidine Phosphorylase
UPLC: Ultra-Performance Liquid Chromatography
