# Supplementary material for: Lysosomal dysfunction and overload of nucleosides in thymidine phosphorylase deficiency of MNGIE
Source: J Transl Med. 2024 May 13;22:449. doi: 10.1186/s12967-024-05275-8 (PMC11089807; doi:10.1186/s12967-024-05275-8)

Supplementary Information

Lysosomal dysfunction and overload of nucleosides in thymidine phosphorylase deficiency of MNGIE

Jixiang Du[1,2,3], Fuchen Liu[2], Xihan Liu[4], Dandan Zhao[2], Dongdong Wang[2], Hongsheng Sun[1,3], Chuanzhu Yan[2,5,6,†] and Yuying Zhao[2,†]

[†]These authors contributed equally to this work.

Correspondence to:

Professor Yuying Zhao

Research Institute of Neuromuscular and Neurodegenerative Disease, Department of Neurology, Qilu Hospital, Shandong University, West Wenhua Street No.107, Jinan, China, 250012

E-mail: zyy72@126.com

Professor Chuanzhu Yan

Research Institute of Neuromuscular and Neurodegenerative Disease, Department of Neurology, Qilu Hospital, Shandong University, West Wenhua Street No.107, Jinan, China, 250012

E-mail: czyan@sdu.edu.cn

Supplementary Table 1 Antibodies used for Western blotting

Supplementary Table 2 Primers used for qPCR

Supplementary Table 3 mtDNA point mutations in the muscle sample of the MNGIE-1 patient

Supplementary Fig. 1 Expression of proteins related to the mitochondrial respiratory chain and mitophagy in the muscles of MELAS patients

(**A, B**) Western blot analyses were performed on muscle tissue samples from m.3243A>G MELAS patients to determine the expression of proteins of the mitochondrial respiratory chain (**A**) and mitochondrial autophagy (**B**) using GAPDH as an internal reference. The numbers on the left indicate the corresponding molecular weight markers (kDa). Data are expressed as mean ± standard deviation and were compiled from three independent experiments. Significance was determined using an unpaired two-tailed Student's t-test: *p <0.05, **p <0.01, ***p <0.001.

Supplementary Fig. 2 The protein content of LAMP1 and SQSTM1 in the muscle sample of patient MNGIE-3

(**A**) Western blot analysis was performed to determine the expression of lysosomal protein LAMP1 and autophagosome protein SQSTM1 in the muscle tissue samples of patient MNGIE-3 and controls, using GAPDH as an internal reference. The numbers on the left indicate the corresponding molecular weight markers (kDa). (**B**) Results of the relative quantification of the Western blot bands. (**C**) Quantification of the GAPDH band density of muscle samples of HC and MNGIE patients. Data are expressed as mean ± standard deviation and were compiled from three independent experiments. Significance was determined using an unpaired two-tailed Student's t-test: *p <0.05, **p <0.01, ***p <0.001.

Supplementary Fig. 3 Magic red and acridine orange staining for MELAS fibroblasts.

(**A**) Fluorescence imaging showed that Magic Red fluorescence intensity in MELAS fibroblasts had no significant changes compared to control cells. (**B**) Fluorescence imaging showed that there were no

significant changes in acridine orange fluorescence intensity in MELAS fibroblasts compared to control cells. (**C**) Quantitative analysis of the relative fluorescence intensity of Magic Red. (**D**) Quantitative analysis of the relative fluorescence intensity of acridine orange. The p-values were calculated using an unpaired two-tailed Student's t-test. Data are expressed as mean ± standard deviation (* $p<0.05$, ** $p<0.01$, *** $p<0.001$).

Supplementary Fig. 4 Lysosomal nucleoside level of 293T cell with thymidine and deoxyuridine treatment

(**A**) 293T cell expressing TMEM192-3×HA was treated with 10 μM thymidine or deoxyuridine for 24 h. After treatment, lysosomes were isolated with Lyso-IP and performed nucleosides measurement with mass spectrometric analysis. The p-values were calculated using an unpaired two-tailed Student's t-test. Data are expressed as mean ± standard deviation (* $p<0.05$, ** $p<0.01$, *** $p<0.001$).

Supplementary Fig. 5 Disturbed mitochondrial homeostasis in cells treated with thymidine and deoxyuridine.

(**A**) Flow cytometric analysis and relative quantification of JC-1 fluorescence intensity were performed on 293T cells treated with CCCP (positive control, 100 μM, 24 hours) and 80 μM thymidine or deoxyuridine for 24 hours. (**B**) ATP content in 293T cells decreased after 48 hours of treatment with 20 μM and 80 μM thymidine or deoxyuridine. (**C**) Relative quantification analysis of mtDNA copy number by qPCR was performed on 293T cells treated with 20 μM and 80 μM thymidine or deoxyuridine for 48 hours. (**D**) The level of mitochondrial superoxide increased in 293T cells after treatment with 20 μM and 80 μM thymidine or deoxyuridine for 48 hours. The p-values were calculated using an unpaired two-tailed Student's t-test. Data are presented as mean ± standard deviation (* $p<0.05$, ** $p<0.01$, *** $p<0.001$).

Supplementary File 9 Uncropped original Western blots

Supplementary Table 1 Antibodies used for Western blotting

Antibody	Host	Manufacturer	Cat No.	Dilution
APG5L/ATG5 antibody	Rabbit	Abcam	ab108327	1:1000
BNIP3L antibody	Rabbit	Proteintech	12986-1-AP	1:1000
cathepsin D (D-7):	Mouse	Santa Cruz	sc-377299	1:500
Cathepsin B Polyclonal antibody	Rabbit	Proteintech	12216-1-AP	1:1000
Citrate synthetase antibody	Rabbit	Abcam	ab96600	1:1000
CTSA	Rabbit	SAB	42724	1:1000
GAPDH antibody	Mouse	Proteintech	10494-1-AP	1:5000
HA-Tag (C29F4) Rabbit mAb	Rabbit	CST	3724	1:1000
LC3B antibody	Rabbit	Abcam	ab51520	1:1000
MT-ATP6 antibody	Rabbit	Abcam	ab192423	1:1000
MT-CYB antibody	Rabbit	Abcam	ab81215	1:1000
ND4 antibody	Rabbit	abclonal	A9941	1:1000
Ndufs4 antidody	Rabbit	Abcam	ab137064	1:1000
p70 S6 Kinase	Rabbit	CST	2708	1:1000
Parkin antibody	Mouse	Abcam	ab77924	1:1000
PD-ECGF (PGF-44C)	Mouse	Santa Cruz	sc-47702	1:1000
PGC1 alpha	Rabbit	Abcam	ab54481	1:1000
PINK1 antibody	Rabbit	Novus Biologicals	BC100-494H	1:1000
Recombinant Anti-NDUFS2 antibody	Rabbit	Abcam	ab192022	1:1000
SQSTM1/p62 antibody	Rabbit	Abcam	ab109012	1:1000
TOMM20 antibody	Mouse	Abcam	ab56783	1:1000
Total OXPHOS Rodent WB Antibody Cocktail	Mouse	Abcam	ab110413	1:1000
Ubiquitin antibody	Rabbit	Abcam	ab7780	1:1000
VDAC1/Porin antibody	Rabbit	Abcam	ab15895	1:1000
β-actin	Mouse	Proteintech	66009-1-Ig	1:5000
HRP Anti-Rabbit IgG H&L	Goat	HuaAn Biotechnology	HA1001	1:5000
HRP Anti-Mouse IgG H&L	Goat	HuaAn Biotechnology	HA1006	1:5000

Supplementary Table 2 Primers used for qPCR

Primers	Sequences (5'to 3')
SERPINA1-F	CAGTGAATAAATGAGGCGTACATCC
SERPINA1-R	GACTGTTTCTCATGCCTCTGGAAAG
SLCO2B1-F	CCTGATGCCTAGGTTTCTTTTCTTG
SLCO2B1-R	GGTCATCTGCCTACCCTAGAAC
mt-ND1-F	TACGGGCTACTACAACCCTTC
mt-ND1-R	ATGGTAGATGTGGCGGGTTT
mt-ND5-F	CATTACTAACAACATTTCCCCCGC
mt-ND5-R	GGCTGTGAGTTTTAGGTAGAGGG
homo-LAMP1-[illegible]	TCTCAGTGAACTACGACACCA
homo-LAMP1-[illegible]	AGTGTATGTCCTCTTCCAAAAGC
h-LAMP2-F	GCACAGTGAGCACAAATGAGT
h-LAMP2-R	CAGTGGTGTGTATGGTGGGT

Supplementary Table 3 mtDNA point mutations in the muscle sample of the MNGIE-1 patient

Gene	Nt position	Nucleotide change	Amino acid change	Report
None	310	C insertion	-	Yes
ND2	4833	A>G	T122A	Yes
ND3	10398	A>G	T114A	Yes
CYTB	15043	G>A	Syn	Yes
CYTB	15784	T>C	Syn	Yes
CYTB	15884	G>A	A380T	No
tRNA-Pro	16183	A>C	-	Yes
tRNA-Pro	16189	T>C	-	Yes

Supplementary Fig. 1

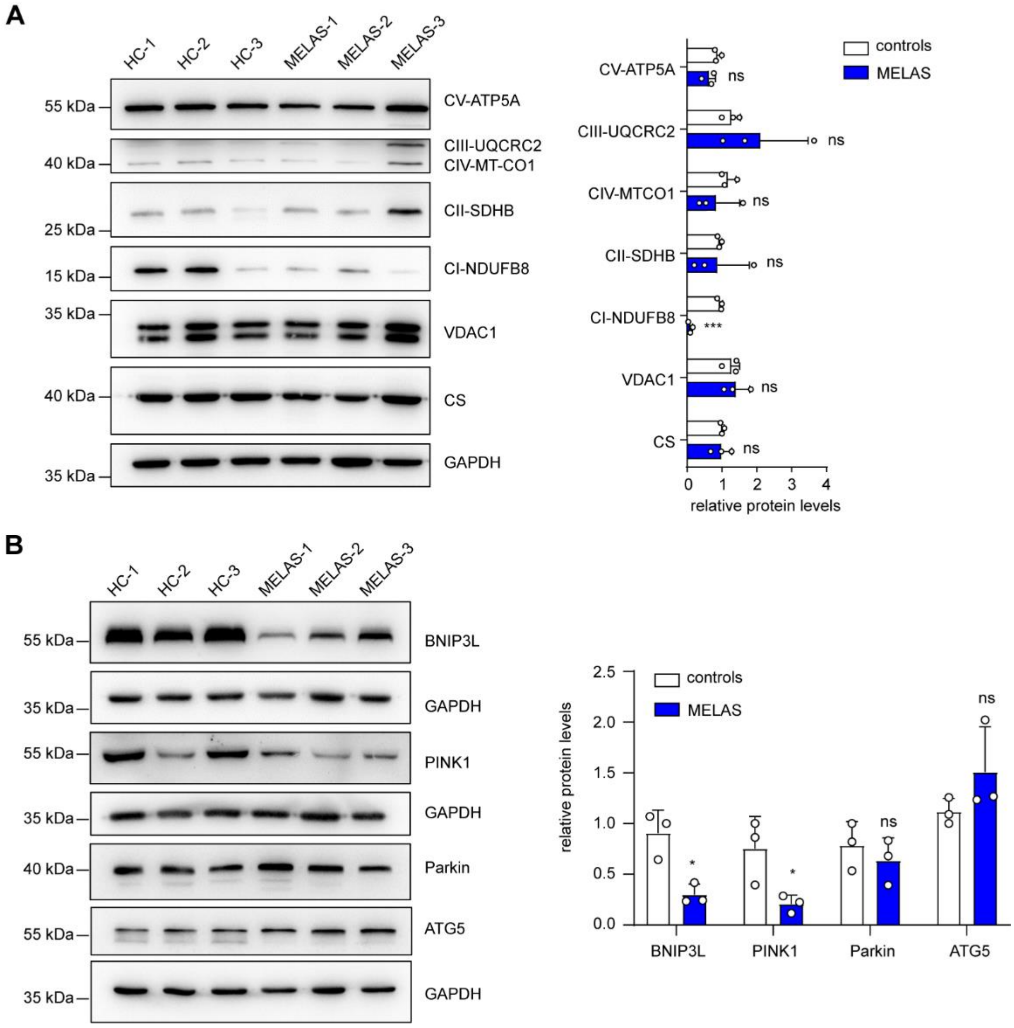

Supplementary Fig. 2

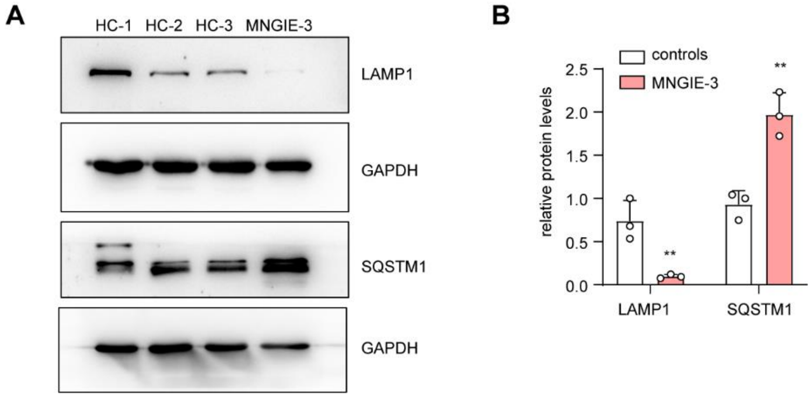

Supplementary Fig. 3

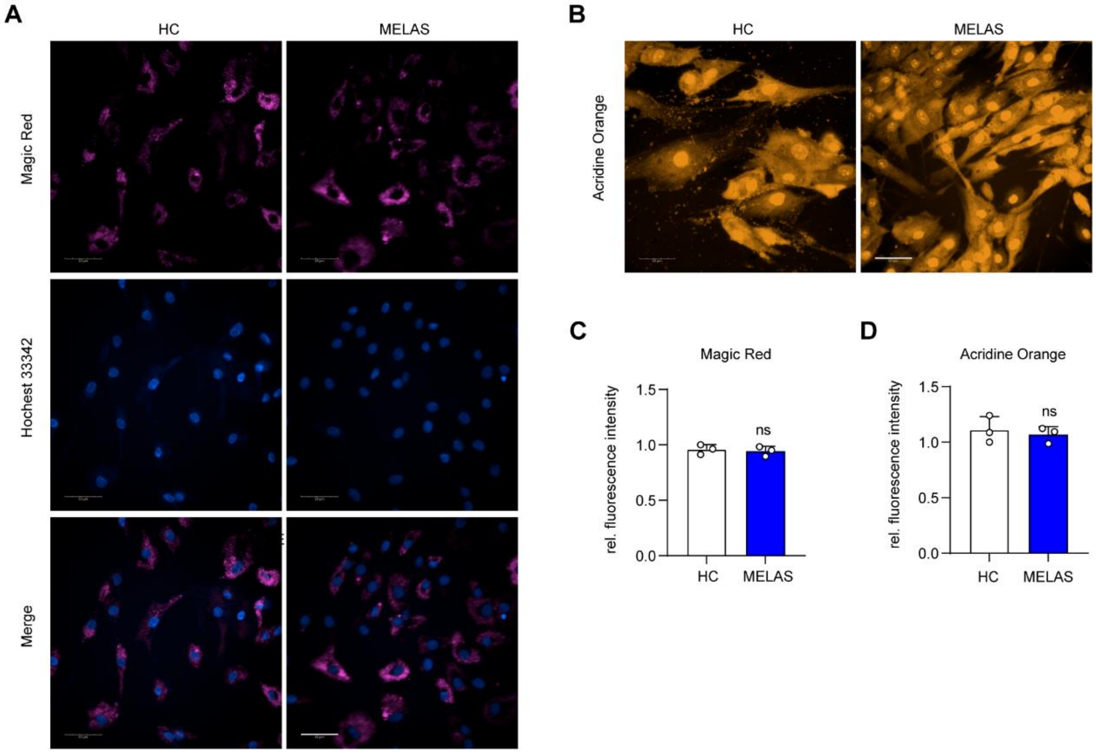

Supplementary Fig. 4

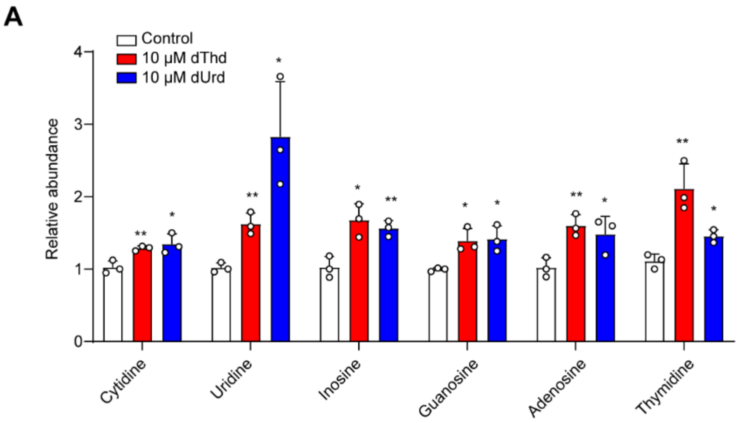

Supplementary Fig. 5

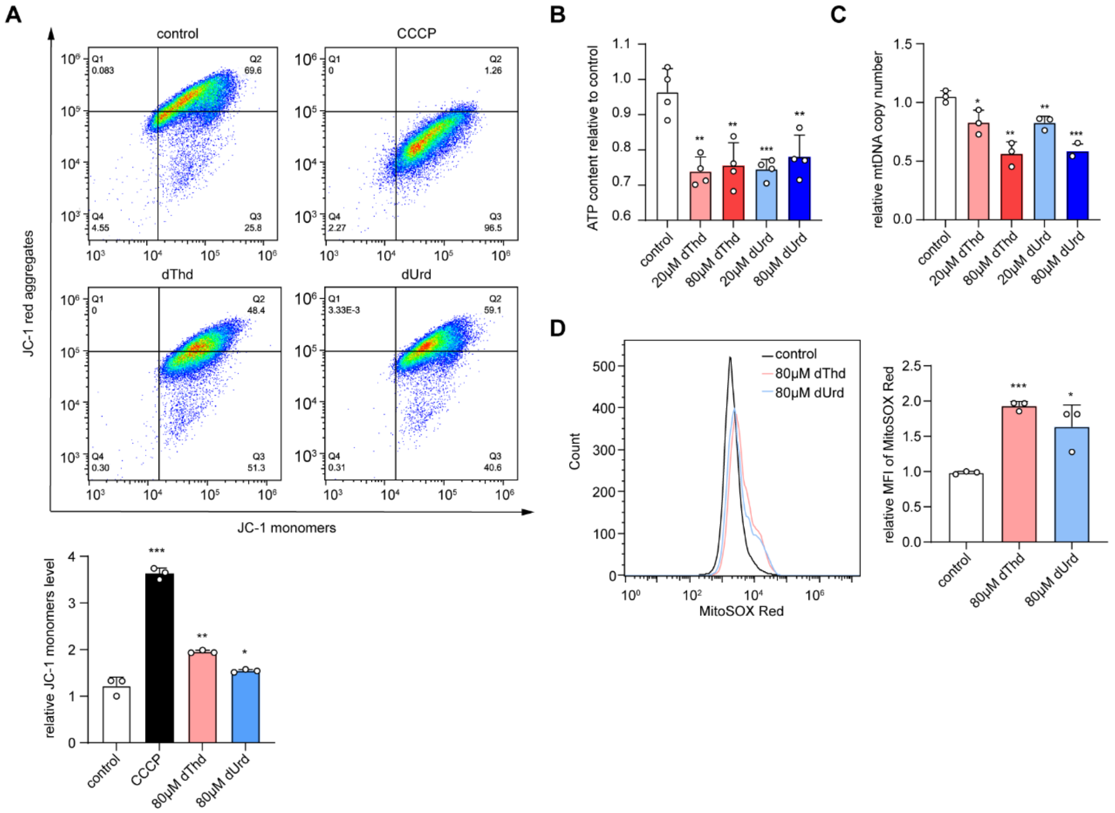

1

Supplementary File 9 Uncropped original Western blots

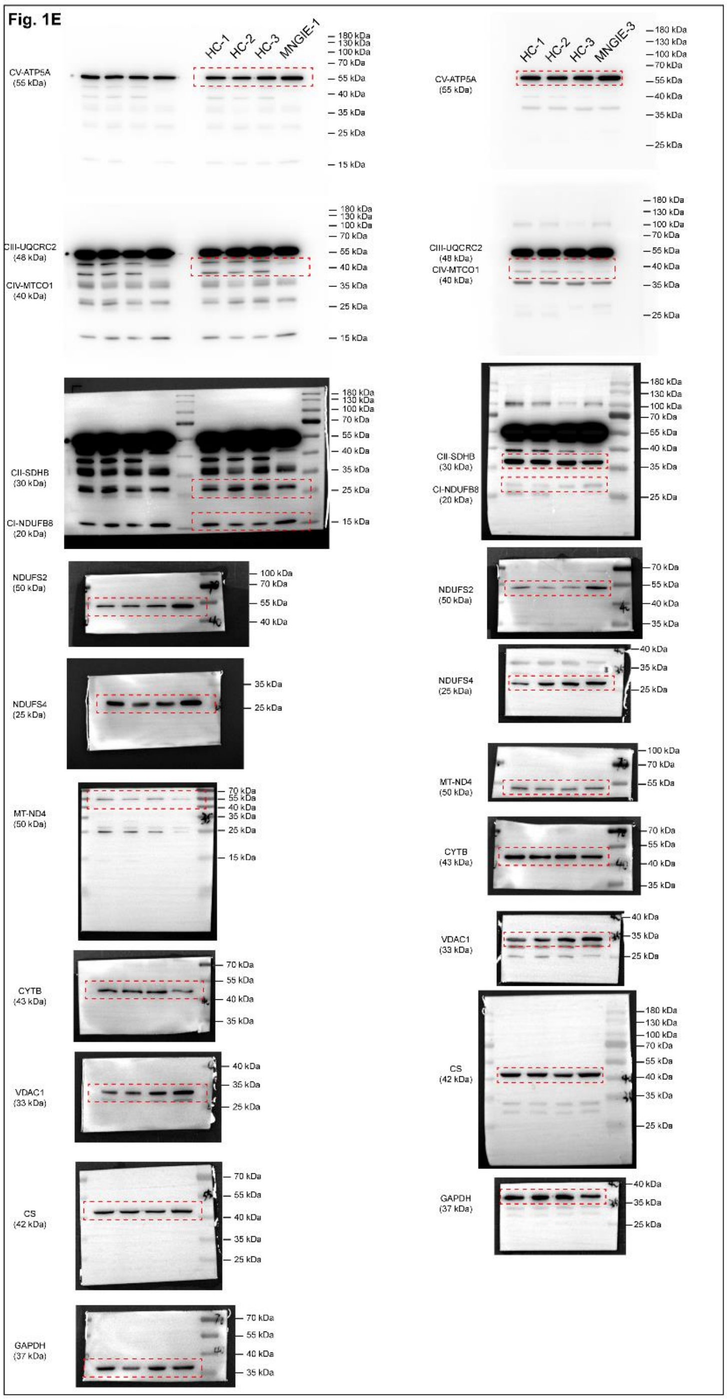

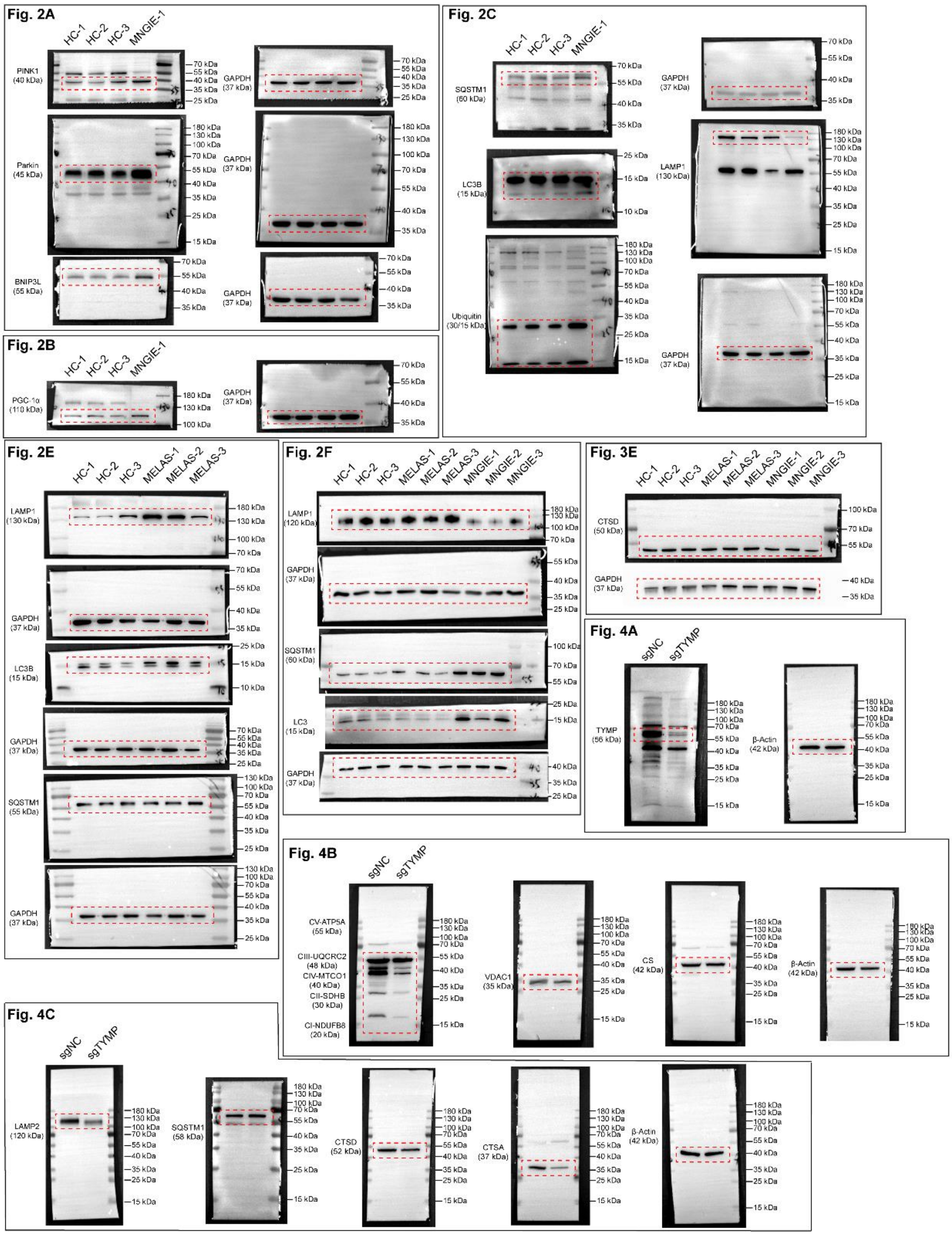
Fig. 2A
HC-1
HC-2
HC-3
MNGIE-1
PINK1 (40 kDa)
Parkin (45 kDa)
BNIP3L (55 kDa)
GAPDH (37 kDa)
Fig. 2B
PGC-1α (110 kDa)
Fig. 2C
SQSTM1 (60 kDa)
LC3B (15 kDa)
Ubiquitin (30/15 kDa)
LAMP1 (130 kDa)
Fig. 2E
MELAS-1
MELAS-2
MELAS-3
LAMP1 (130 kDa)
LC3B (15 kDa)
SQSTM1 (55 kDa)
Fig. 2F
MNGIE-2
MNGIE-3
LAMP1 (120 kDa)
SQSTM1 (60 kDa)
LC3 (15 kDa)
Fig. 3E
CTSD (50 kDa)
Fig. 4A
sgNC
sgTYMP
TYMP (56 kDa)
β-Actin (42 kDa)
Fig. 4B
CV-ATP5A (55 kDa)
CIII-UQCRC2 (48 kDa)
CIV-MTCO1 (40 kDa)
CII-SDHB (30 kDa)
CI-NDUFB8 (20 kDa)
VDAC1 (35 kDa)
CS (42 kDa)
Fig. 4C
LAMP2 (120 kDa)
SQSTM1 (58 kDa)
CTSD (52 kDa)
CTSA (37 kDa)

Fig. 4E

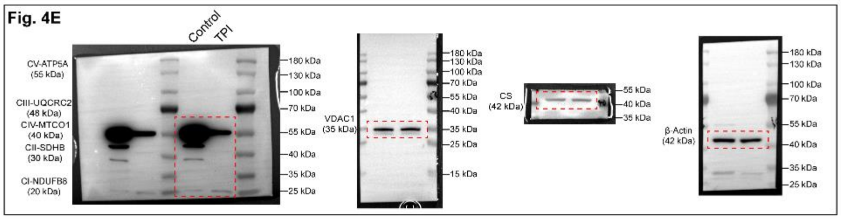

Fig. 4G

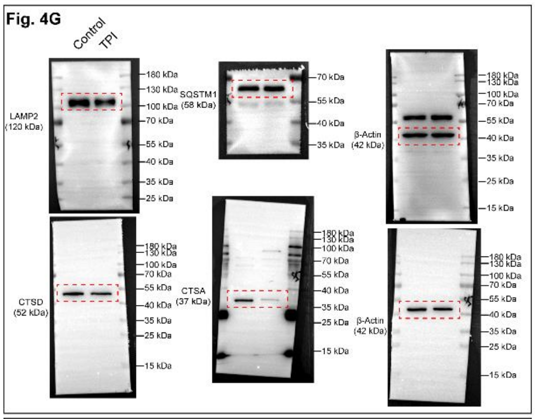

Fig. 5B

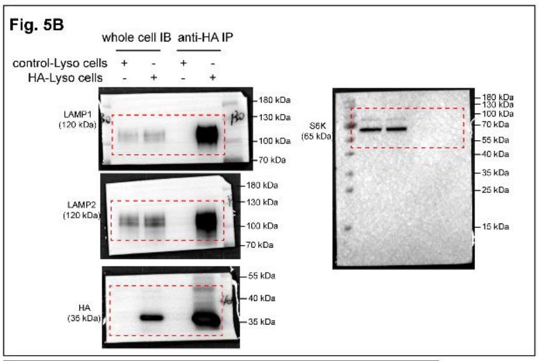

Fig. 6B

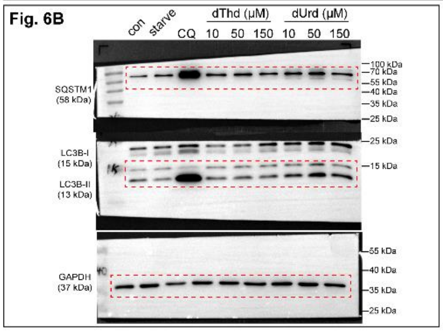

Fig. 6C

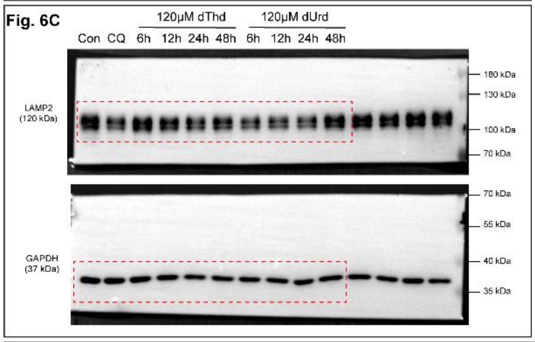

Fig. 6D

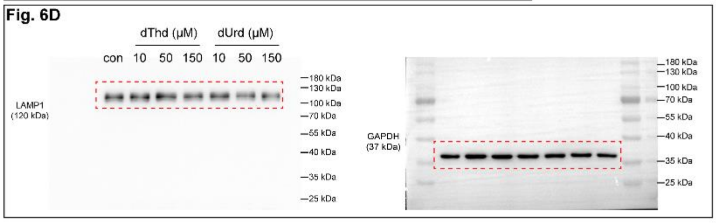

Supplementary Fig. S1A

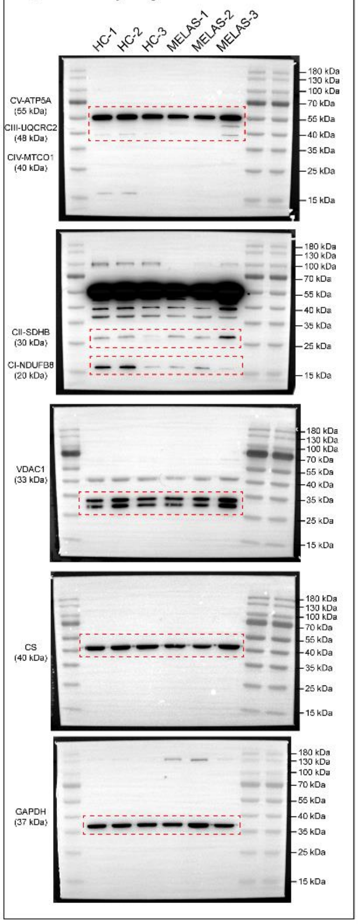

Supplementary Fig. S1B

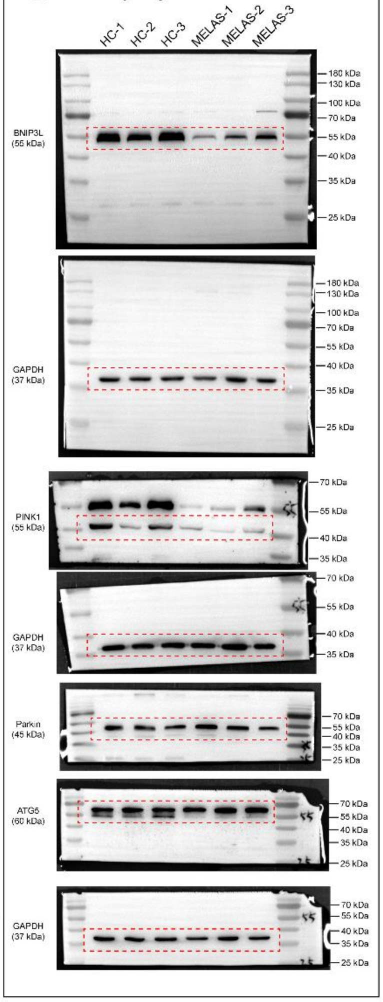

Supplementary Fig. S2A

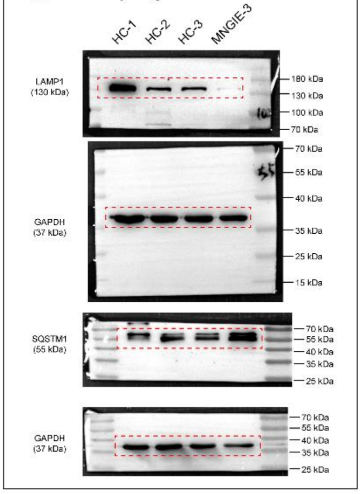

Supplement: Supplementary file 1 — Supplementary Material 1 [file 12967_2024_5275_MOESM1_ESM.pdf]
